# Supplementary material for: Inferring the age and environmental characteristics of fossil sites using citizen science
Source: PLoS One. 2023 Apr 17;18(4):e0284388. doi: 10.1371/journal.pone.0284388 (PMC10109468; doi:10.1371/journal.pone.0284388)
Supplement: S1 Fig — (PDF) [file pone.0284388.s001.pdf]

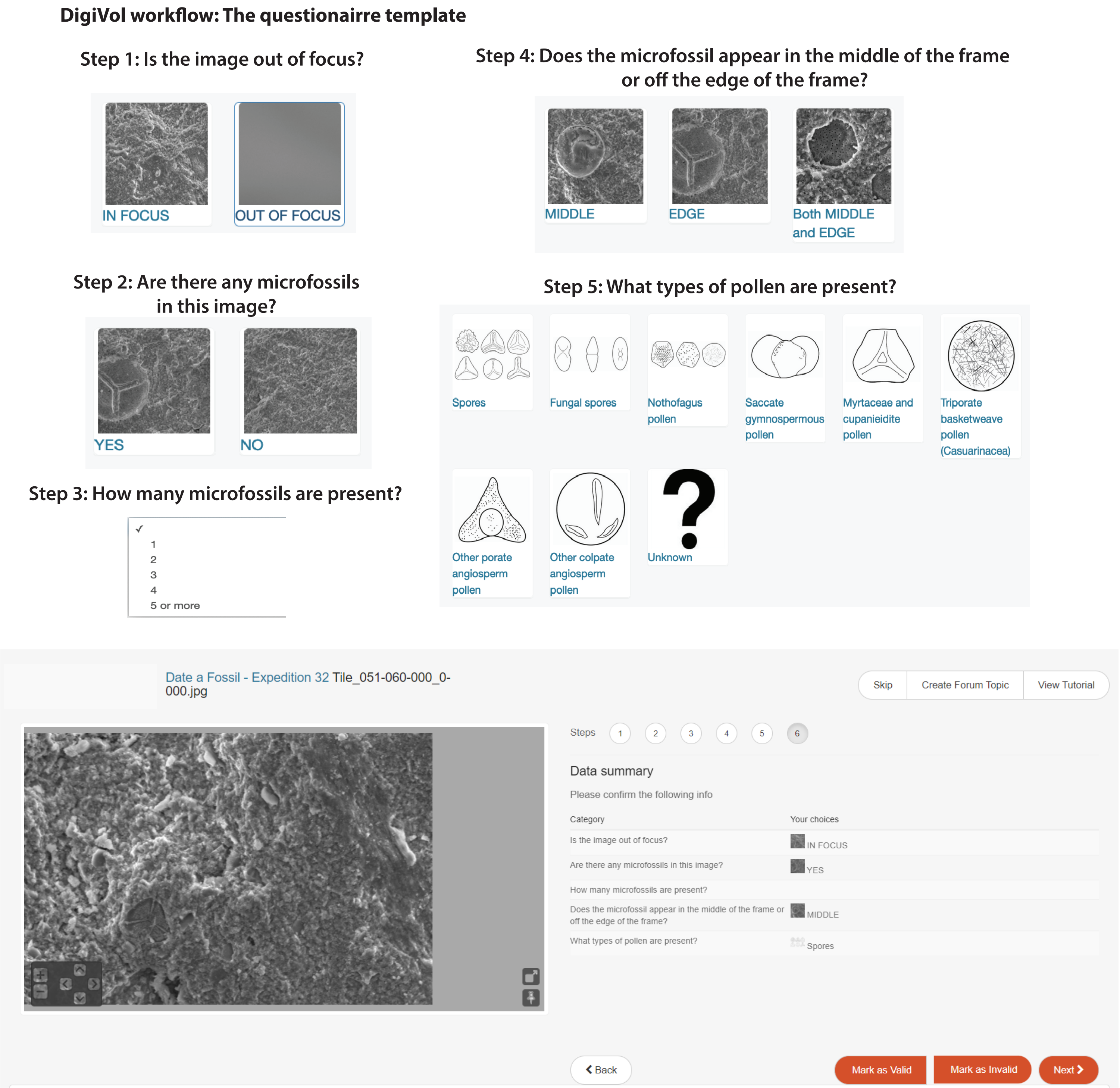

S1 Figure. Screenshots of the questionnaire template for the 'Date a Fossil Project' hosted on DigiVol.
