# Supplementary material for: Inferring the age and environmental characteristics of fossil sites using citizen science
Source: PLoS One. 2023 Apr 17;18(4):e0284388. doi: 10.1371/journal.pone.0284388 (PMC10109468; doi:10.1371/journal.pone.0284388)
Supplement: S2 Fig — Pollen images from page 5 (Fungal spore, Nothofagus pollen), page 8 (Myrtaceae and cupanieidite pollen), and page 10 (angiosperm pollen) are modified from McCurry et al., 2022. (PDF) [file pone.0284388.s002.pdf]

S2 Figure. Training manual and microfossil identification guide for citizen scientists engaging with 'Date a Fossil' on DigiVol. Pollen images from page 5 (Fungal spore, Nothofagus pollen), page 8 (myrtaceae and cupanieidite pollen), and page 10 (angiosperm pollen) are modified from McCurry et al., 2022.

IDENTIFICATION GUIDE – CROWDSOURCING THE AGE OF FOSSILS

Background

This project is a collaboration between the Australian Museum and The University of Canberra. We are seeking your help to determine the age, and environmental characteristics of Australian fossil sites. The images uploaded to DigiVol have been automatically collected using a scanning electron microscope. Please answer the questions for each image. The resulting data will provide scientists with information about the variety and relative abundance of the microfossils in fossil sites.

The importance of understanding the definition of each criteria

Question 1 – Is the image out of focus?

The images are automatically collected from a Scanning Electron Microscope and can sometimes be out of focus.

If it is impossible to tell what is in the image, please select "OUT OF FOCUS". If the image is clear enough to make out the microfossils please select "IN FOCUS".

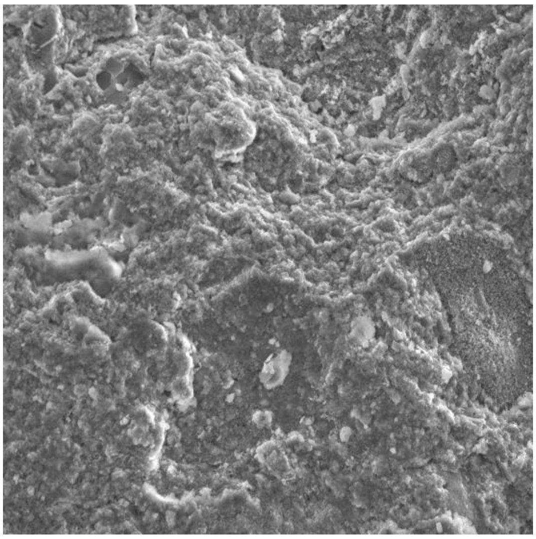

IN FOCUS

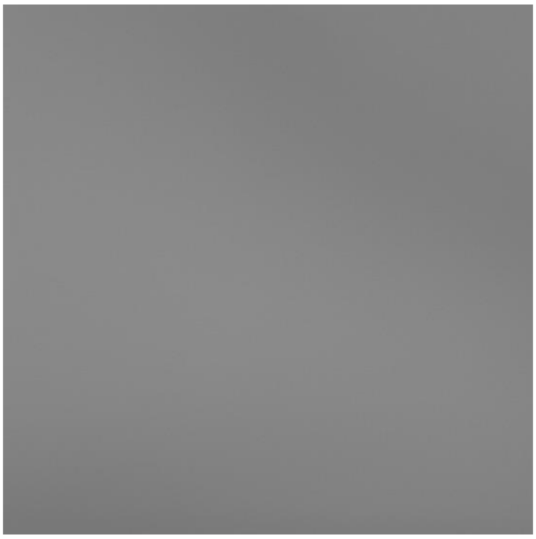

OUT OF FOCUS

If the image is **out of focus**, please proceed to **STEP 6** and submit your task. If it is in focus, please proceed to question 2.

Question 2 - Are there any microfossils in this image?

Please answer “YES” or “NO”

Most images will not have anything in them. If your image **does not** have any microfossils in it, then please proceed to **STEP 6** and submit your task.

Below is an example of an image without any microfossils present.

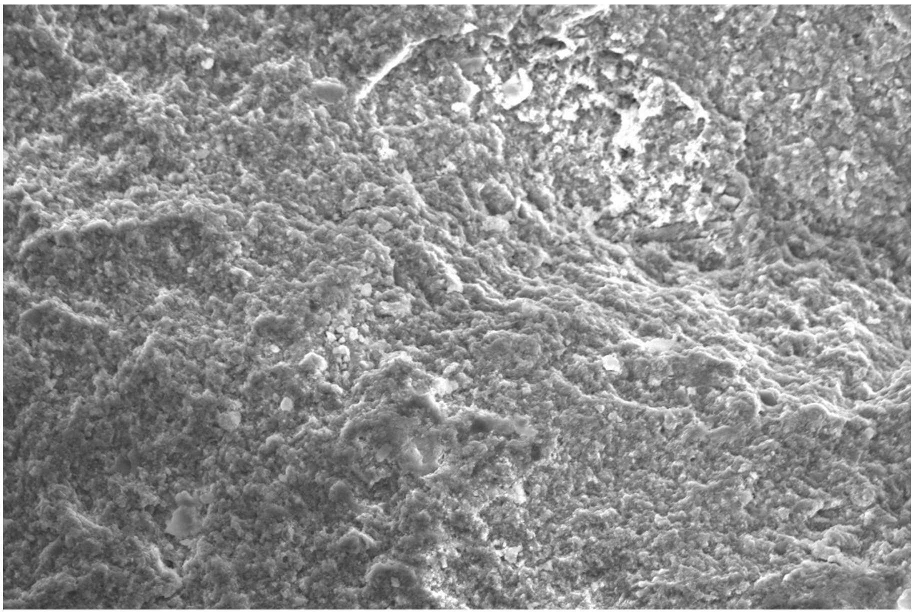

Question 3 - How many microfossils are present?

Sometimes multiple fossils can appear in one image. Please enter the number of microfossils present.

For example, here is an image containing two pollen grains (*Nothofagidites*).

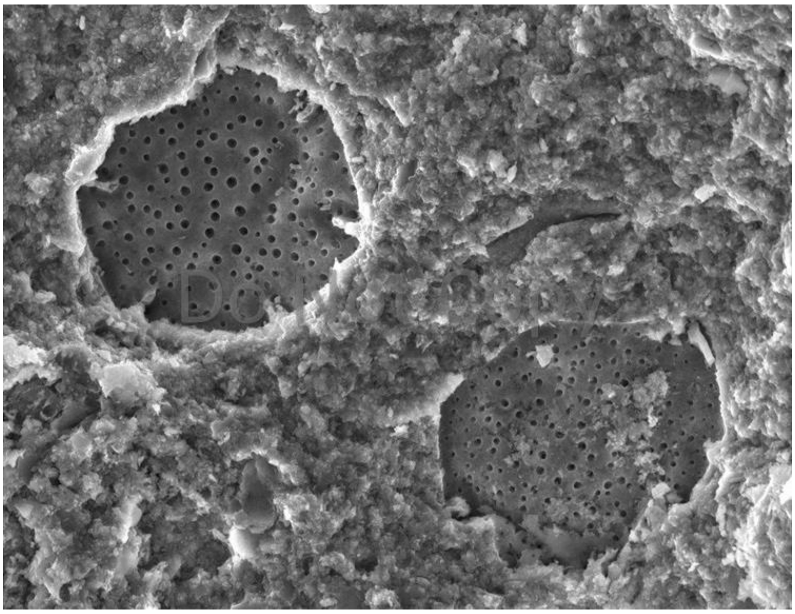

Question 4 - Does the microfossil appear in the middle of the frame or off the edge of the frame?

Please select “MIDDLE” or “EDGE” or “Both MIDDLE and EDGE” depending on where the microfossil is in the frame of the image.

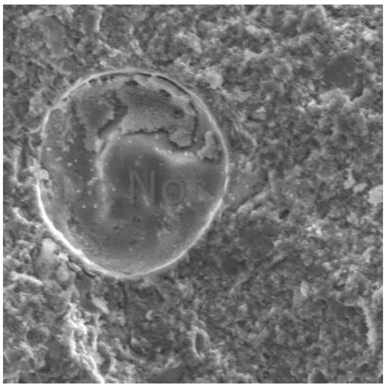

MIDDLE

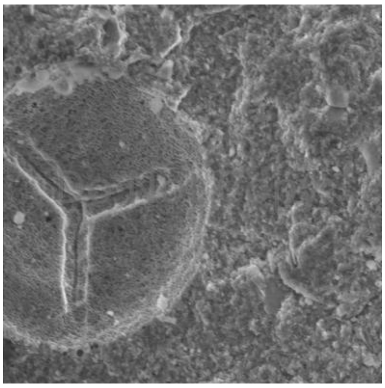

EDGE

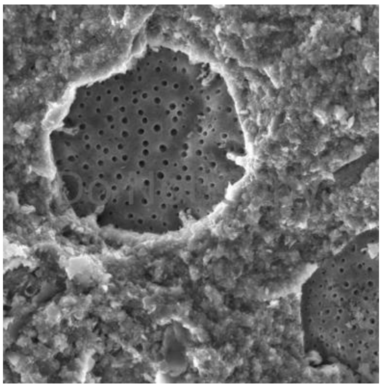

Both MIDDLE and EDGE

Question 5 – What types of pollen/spores are present?

Please select the category of microfossil that matches the microfossils in your image.

If multiple categories of microfossils are present you can select multiple categories.

Categories are as below.

Spores

Distinguishing features:

- **Trilete scar (Y-shaped).**

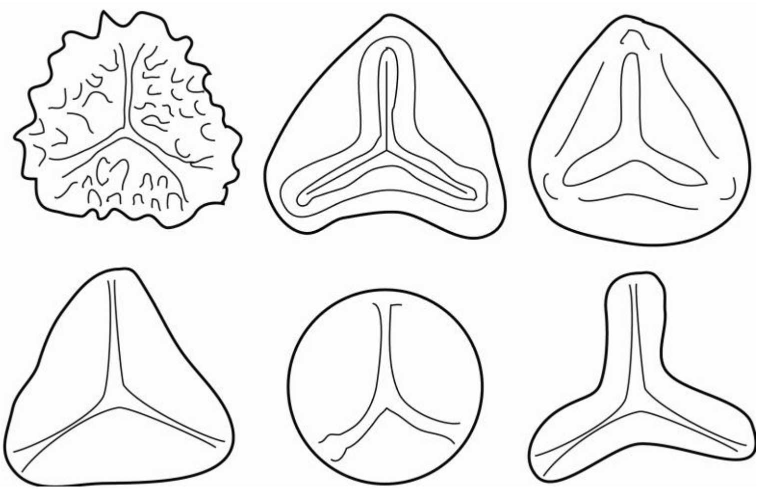

Below are some examples of spores.

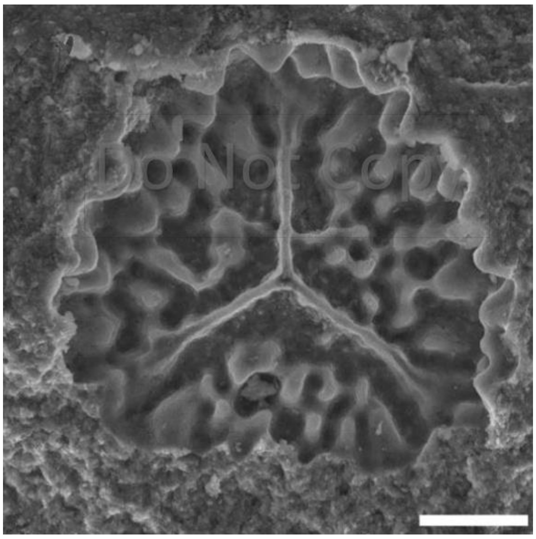

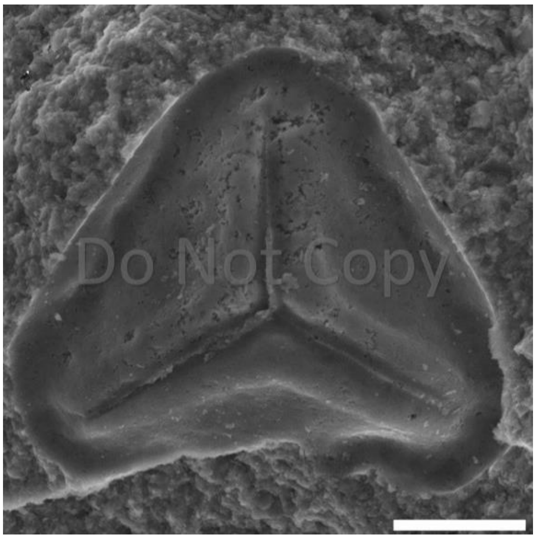

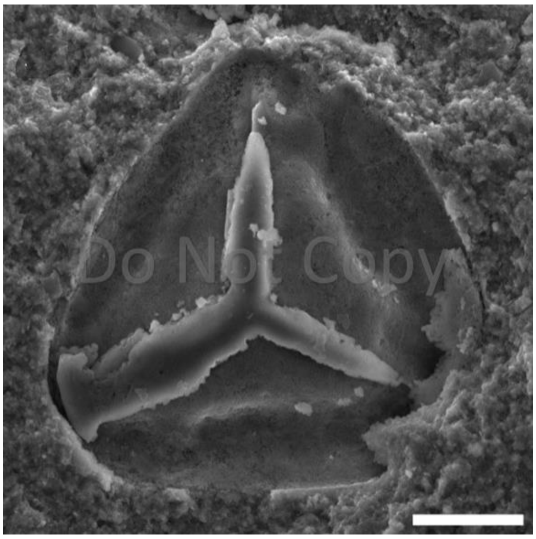

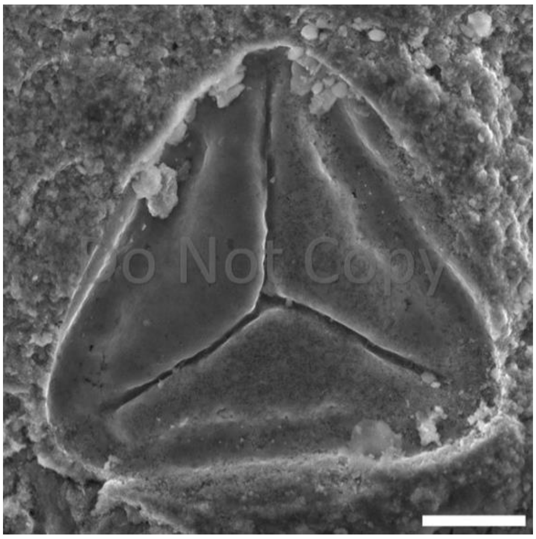

Fungal Spores

Distinguishing features:

- **Ovoid or two sided.**
- **Usually much smaller than trilete spores.**
- **No obvious slits or pores.**
- **Usually smooth.**

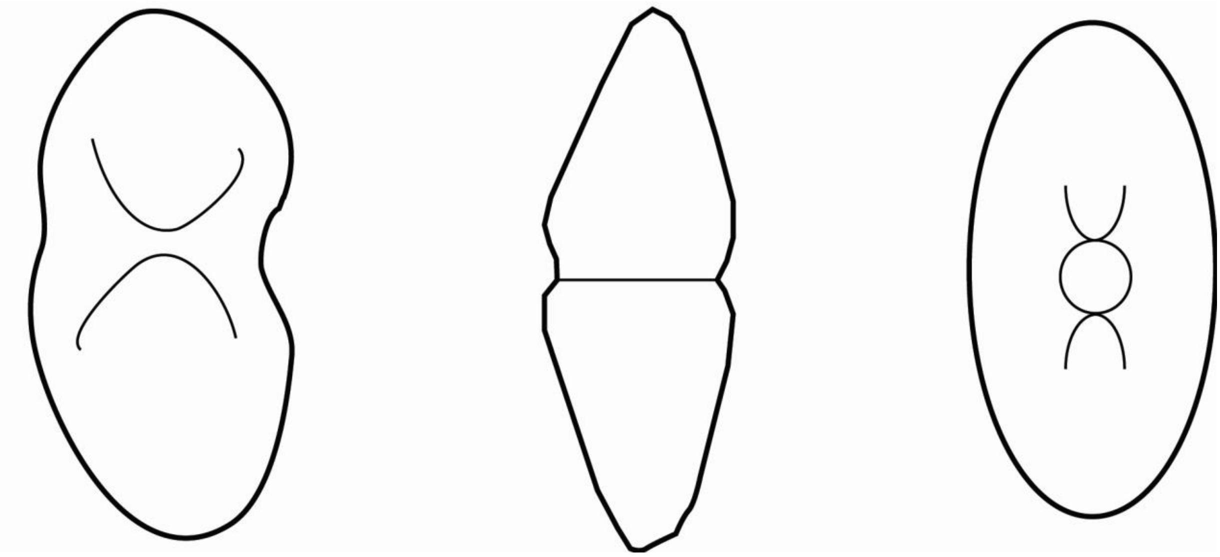

Below are some examples of fungal spores.

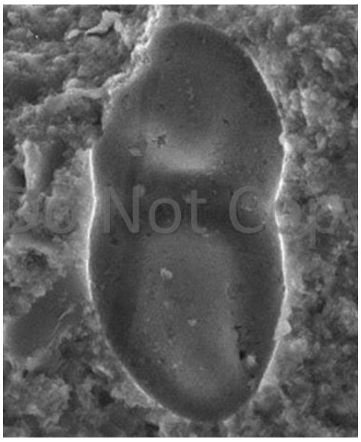

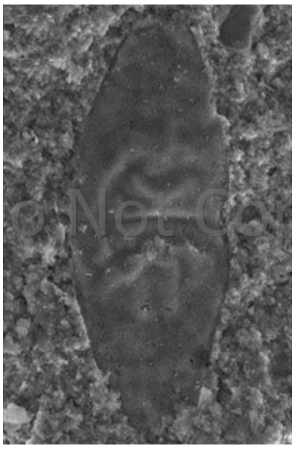

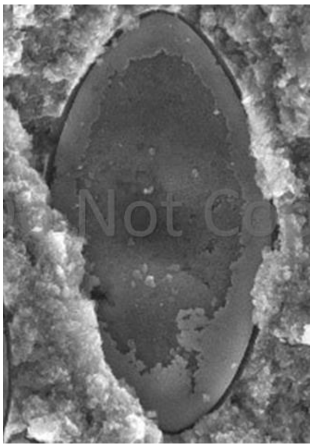

Nothofagus pollen

Distinguishing features:

- **Disc-shaped.**
- **Distinctive pits.**
- **Slits at periphery.**

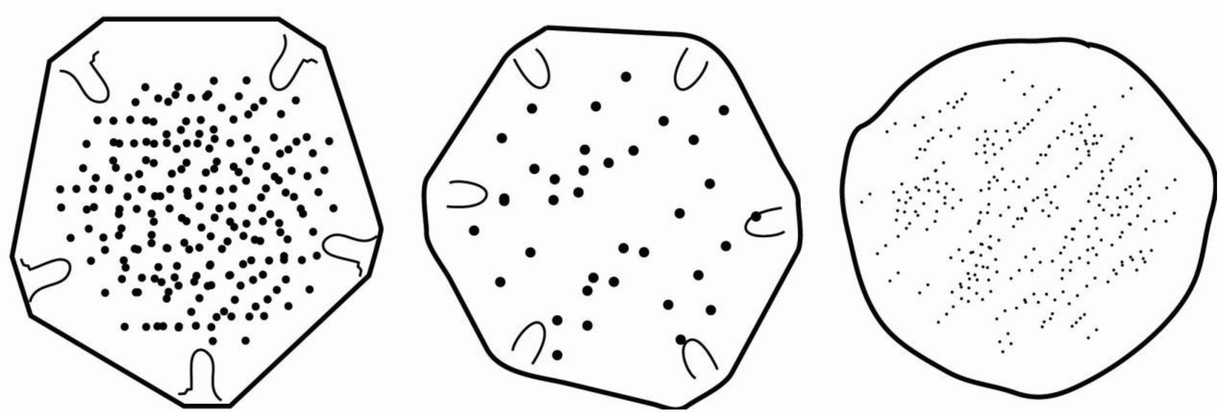

Below are some examples of *Nothofagidites*.

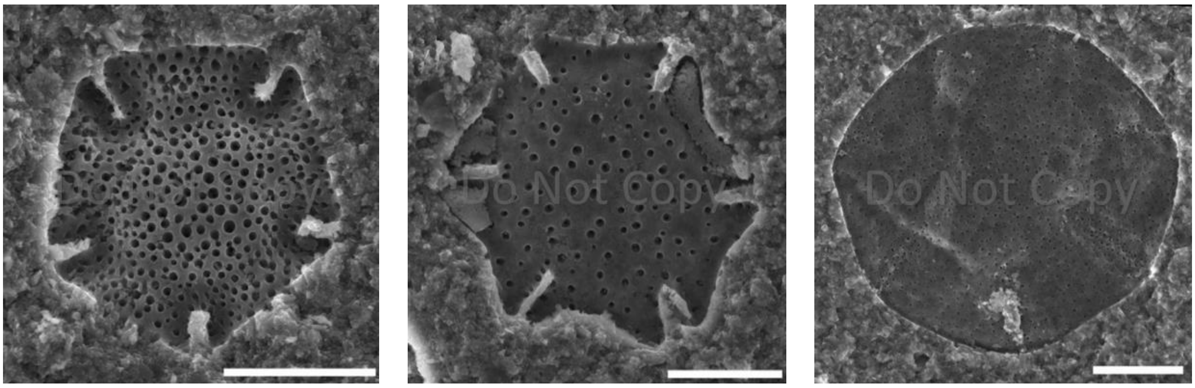

Saccate gymnospermous pollen

Distinguishing features:

- **Pollen with 2 air sacs.**
- **Mickey Mouse-like outline.**

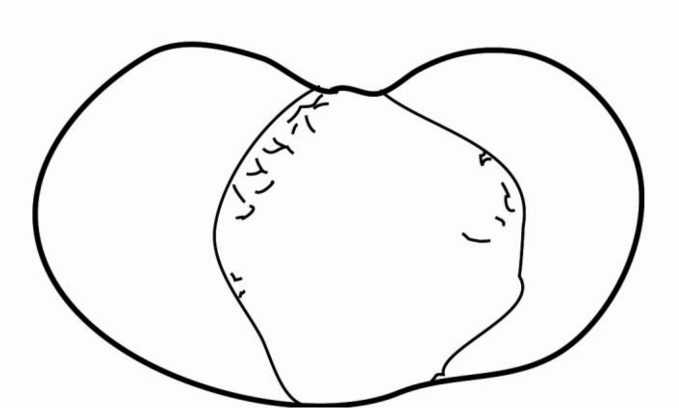

Below is an example of a saccate gymnospermous pollen.

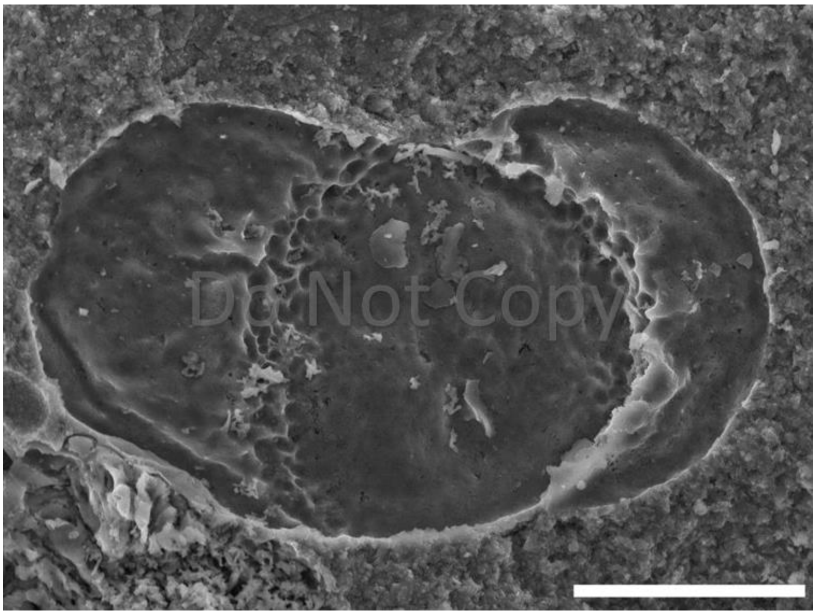

Myrtaceae and cupanieidite pollen

Distinguishing features:

- **Triangular-shaped pollen with gaping Y mark extending to angles.**
- **Much smaller than most spores.**
- **With blunt edges at angles of triangular outline.**

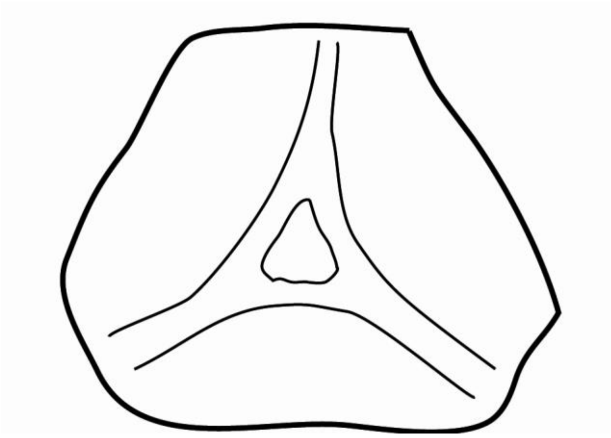

Below is an example of myrtaceae and cupanieidite pollen.

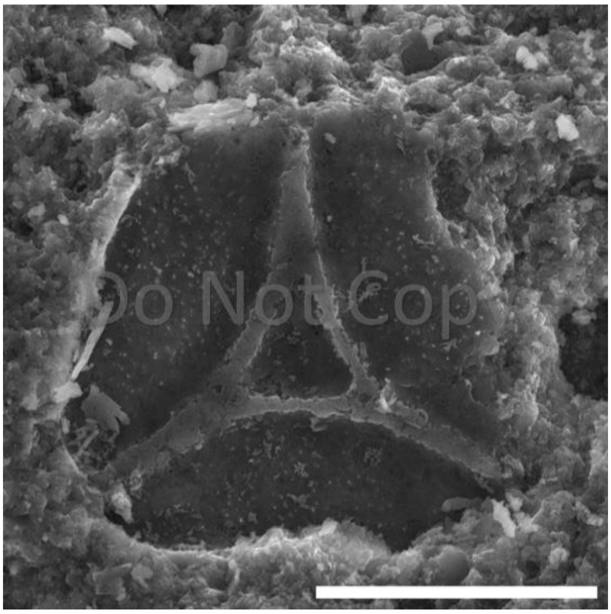

Triporate basketweave pollen (Casuarinacea)

Distinguishing features:

- **Three pores.**
- **“Basketweave” pattern.**
- **Triangular outline with convex sides.**

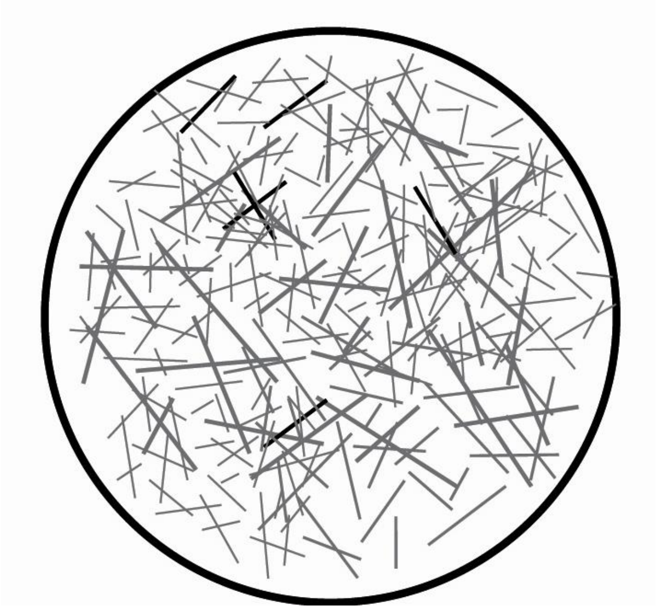

Below is an example of a triporate basket weave pollen (Casuarinacea).

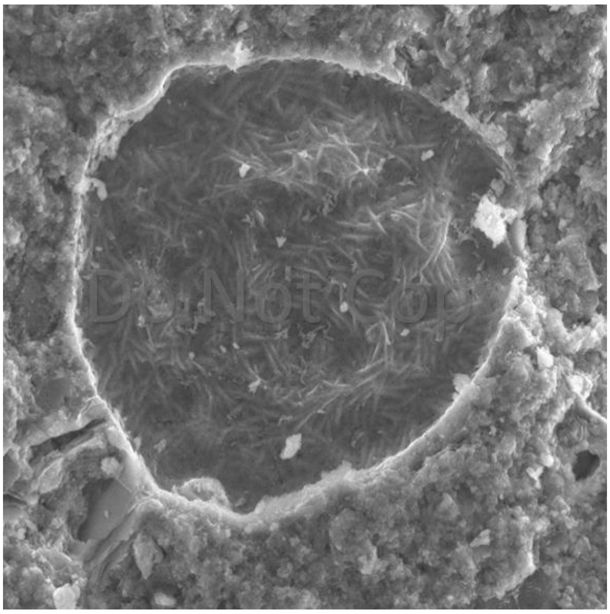

Porate angiosperm pollen

Distinguishing features:

- **Triporate.**
- **Triangular with straight or concave sides.**
- **Reticulate or spiky pattern.**

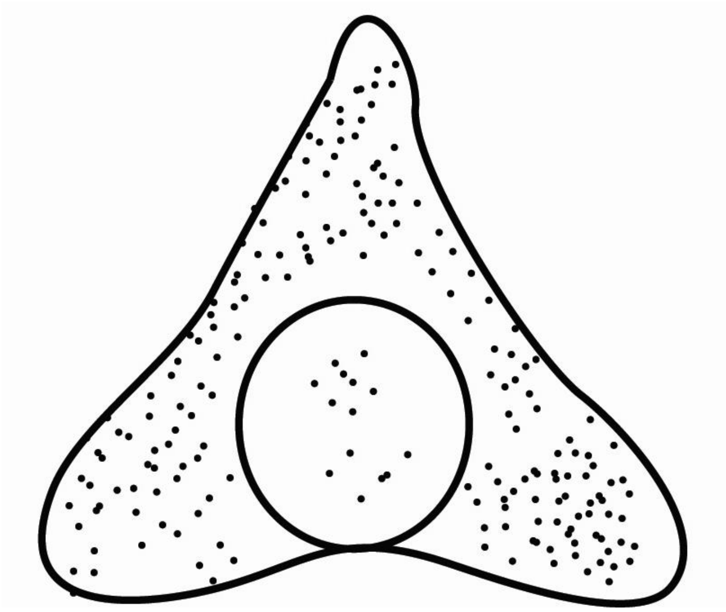

Below is an example of a porate angiosperm pollen.

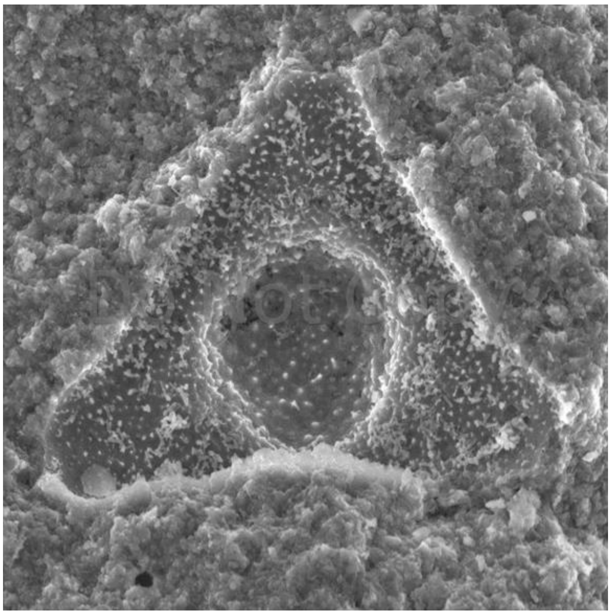

Colpate angiosperm pollen

Distinguishing features:

- **Colpate (slits).**
- **Usually rounded (subcircular outline).**
- **Spiky or other pattern or sometimes no obvious pattern.**

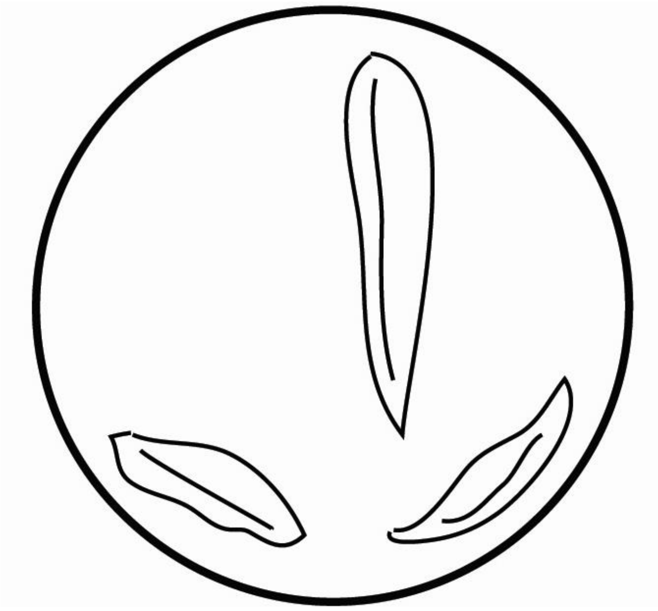

Below is an example of a colpate angiosperm pollen.

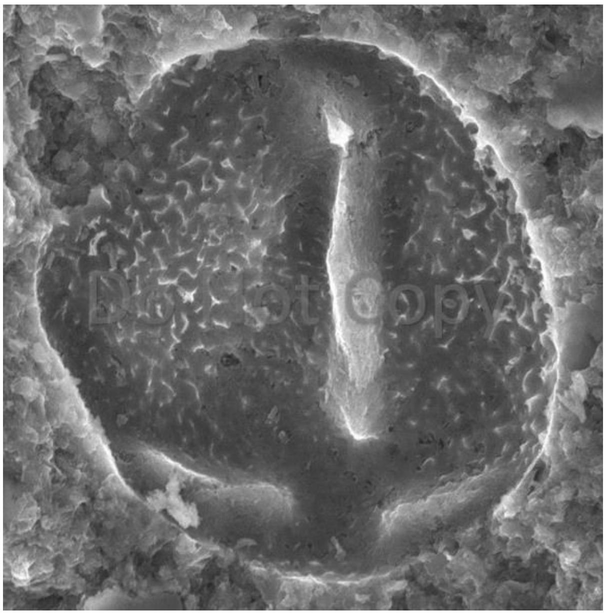

Unknown/Miscellaneous

Distinguishing features:

- **A wide variety of other microfossils can also be found within the images.**
- **Common examples include small insects, spiders and mites as well as plant remains, cyanobacteria and diatoms.**
- **If something is present but does not fit any of the categories, please categorise it as miscellaneous.**

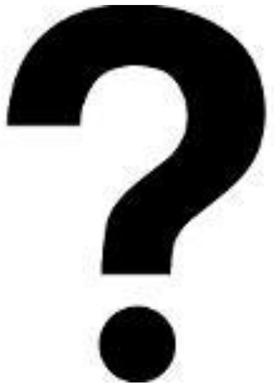

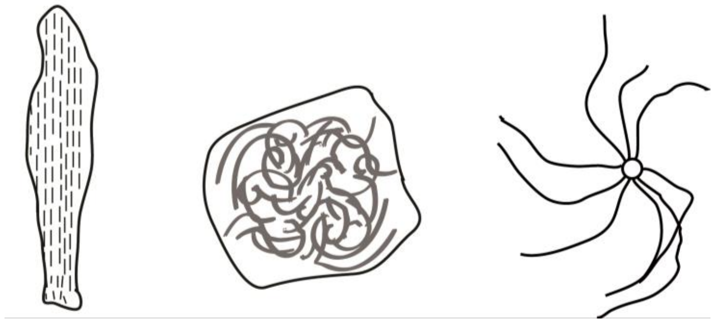

Below are some examples of miscellaneous structures.

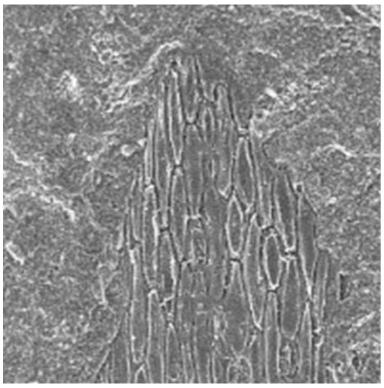

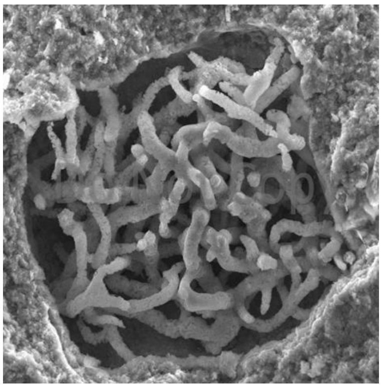

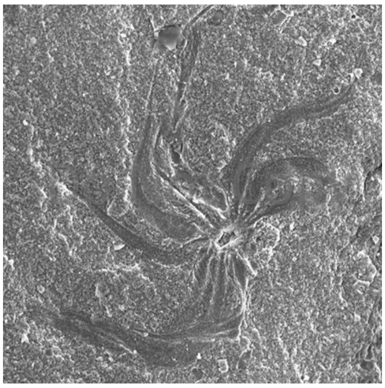

Approximate relative size of various microfossils.

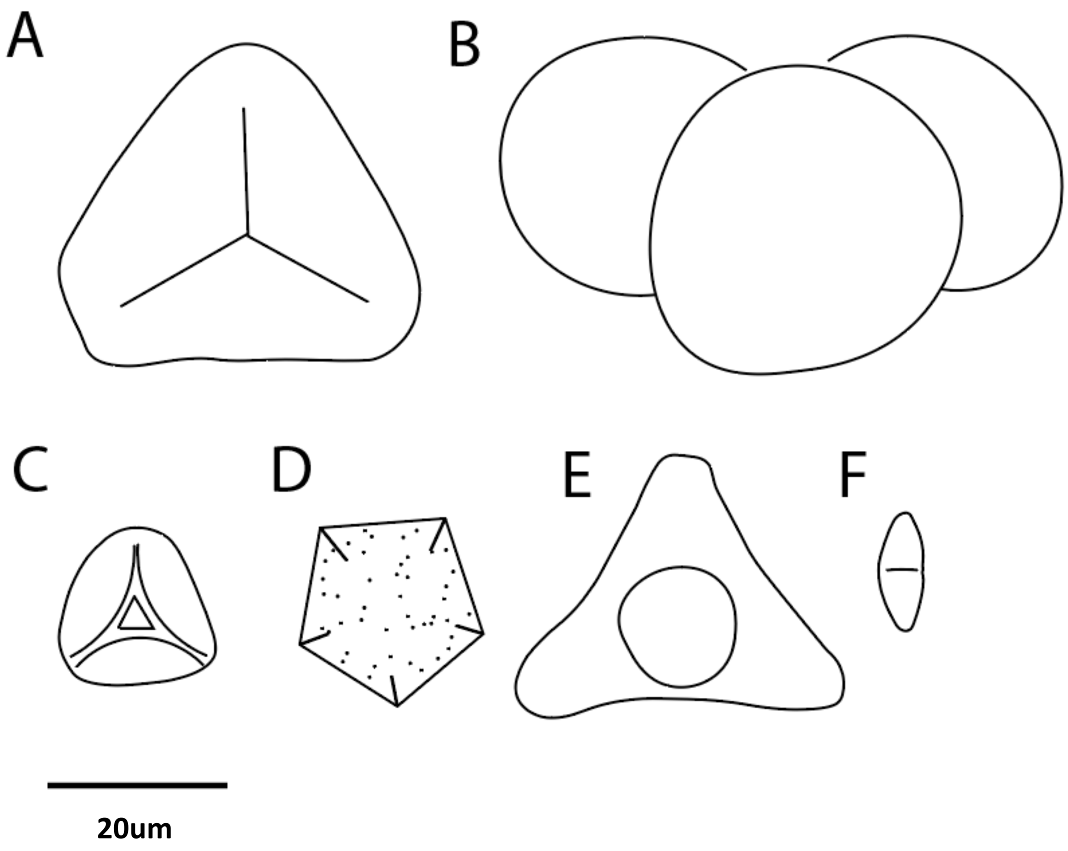

Approximate relative size of microfossils. A: fern spores B: saccate gymnospermous pollen, C: Myrtaceidites and Cupanieidites pollen D: Nothofagidites pollen E: triporate angiosperm pollen and F: a fungal spore. Note that fern spores are some of the larger microfossils.

PLEASE NOTE:

The images displayed in the Date a Fossil expeditions are **120um** in width.
