# Supplementary material for: Inferring the age and environmental characteristics of fossil sites using citizen science
Source: PLoS One. 2023 Apr 17;18(4):e0284388. doi: 10.1371/journal.pone.0284388 (PMC10109468; doi:10.1371/journal.pone.0284388)
Supplement: S3 Fig — Edited (a-k) and unedited (a-k) micrographs of pollen and spore examples from McGraths Flat. At the centre is Fig 6 from the manuscript, which is surrounded by outset images of the corresponding original unedited SEM micrographs that were viewed by citizen scientists. (a) Fungal spore. (b) Fern spore. (c) Nothofagidites. (d) Gymnospermous saccate pollen. (e) Myrtaceae/Cupanieae pollen. (f) Triporate pollen. (g) Porate angiosperm pollen. (h) Araucariaceae, Gymnosperm pollen. (i) Angiosperm col(por)ate pollen; Quintinia. (i) Angiosperm colporate pollen; Margocolporites vanwijhei. (k) Angiosperm colpate pollen. Dashed lines indicate the overlap/stitching between images. Arrows indicate the microfossils. Scale bars, 15 μm. Field of view for all outset unedited images, 120 μm. (PDF) [file pone.0284388.s003.pdf]

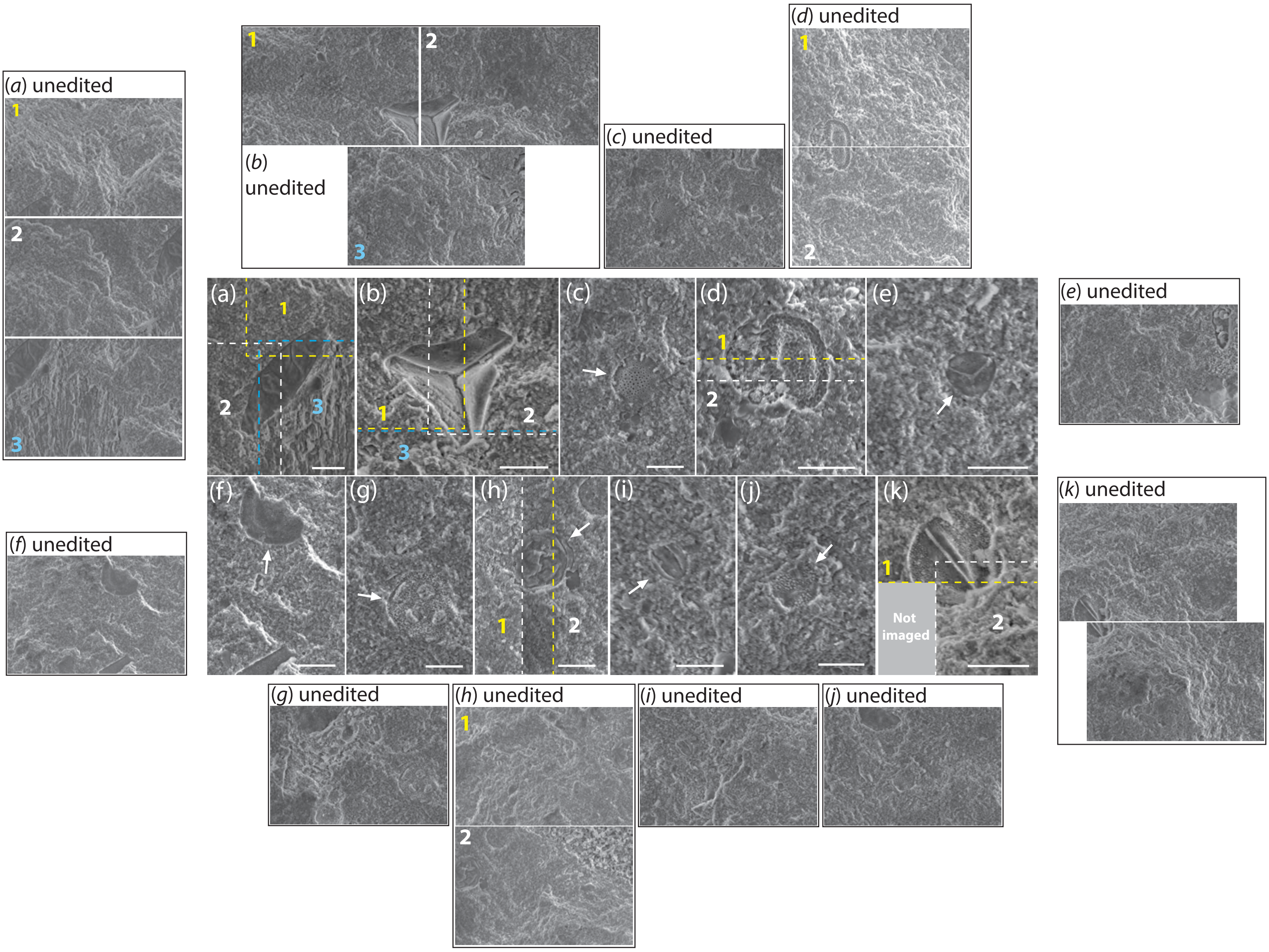

S3 Figure. Edited (a-k) and unedited (a-k) micrographs of pollen and spore examples from McGraths Flat. At the centre is Figure 6 from the manuscript, which is surrounded by outset images of the corresponding original unedited SEM micrographs that were viewed by citizen scientists. (a) Fungal spore. (b) Fern spore. (c) *Nothofagidites*. (d) Gymnospermous saccate pollen. (e) Myrtaceae/Cupanieae pollen. (f) Triporate pollen. (g) Porate angiosperm pollen. (h) Araucariaceae, Gymnosperm pollen. (i) Angiosperm col(por)ate pollen; *Quintinia*. (j) Angiosperm colporate pollen; *Margocolporites vanwijhei*. (k) Angiosperm colpate pollen. Dashed lines indicate the overlap/stitching between images. Arrows indicate the microfossils. Scale bars, 15 µm. Field of view for all outset unedited images, 120 µm.
