# Supplementary material for: Inferring the age and environmental characteristics of fossil sites using citizen science
Source: PLoS One. 2023 Apr 17;18(4):e0284388. doi: 10.1371/journal.pone.0284388 (PMC10109468; doi:10.1371/journal.pone.0284388)
Supplement: S1 Table — Total number of images in which volunteers reached agreement or did not reach agreement on the questionnaire template. Total number of images that needed expert review, as well as images verified to contain pollen/spores and final pollen/spore counts. (PDF) [file pone.0284388.s005.pdf]

S1 Table. Breakdown of total number of images transcribed by volunteers and expert reviewed. Total number of images in which volunteers reached agreement or did not reach agreement on the questionnaire template. Total number of images that needed expert review, as well as images verified to contain pollen/spores and final pollen/spore counts.

	Volunteers	271
	**Total images transcribed**	**25200**
Three in agreement, no microfossil	Three in agreement, total images	23612
	Three in agreement, no microfossil	23481
	Three in agreement, no microfossil - other disputed, expert reviewed images	**2473**
	Three in agreement, no microfossil - other disputed, expert verified images	62
	**Total pollen and spore count**	**49**
Three in agreement microfossil	Three in agreement - unknown microfossils	33
	Three in agreement - pollen or spore	98
	Three in agreement - total images (with pollen, spore or unknown), expert reviewed images	**131**
	Three in agreement, correct ID of pollen, spore or unknown	111
	Three in agreement, incorrect ID of pollen, spore or unknown	20
	Three in agreement - pollen or spore, expert verified images	95
	**Total pollen and spore count**	**91**
No agreement	No agreement, total, expert reviewed images	**1588**
	No agreement - contains pollen or spore, expert verified images	275
	**Total pollen and spore count**	**243**
	**Total images expert reviewed**	**4192**
	**Final pollen and spore count (300 identified taxa + 83 unidentified taxa)**	**383**
