# Supplementary material for: Inferring the age and environmental characteristics of fossil sites using citizen science
Source: PLoS One. 2023 Apr 17;18(4):e0284388. doi: 10.1371/journal.pone.0284388 (PMC10109468; doi:10.1371/journal.pone.0284388)
Supplement: S2 Table — (PDF) [file pone.0284388.s006.pdf]

S2 Table. Total pollen and spore counts (expert verified) from 25,200 SEM images analysed by citizen scientists and palynomorph density/cm2.

Pollen and spore	count	Density (cm^2)
Gymnosperm, other	2	1.1
Angiosperm, other	3	1.6
Spores	29	15.7
Fungal spores	60	32.4
Nothofagus Pollen	134	72.4
Saccate gymnospermous pollen	20	10.8
Myrtaceae & cupanieidite pollen	20	10.8
Triporate (Casuarinacea)	14	7.6
Porate angiosperm pollen	2	1.1
Colpate angiosperm pollen	16	8.6
Total identified	300	162.2
Total possible pollen or spore (indet)	83	44.9
Total	383	207.0
