# Supplementary material for: Inferring the age and environmental characteristics of fossil sites using citizen science
Source: PLoS One. 2023 Apr 17;18(4):e0284388. doi: 10.1371/journal.pone.0284388 (PMC10109468; doi:10.1371/journal.pone.0284388)
Supplement: Attachment — Submitted filename: PONE-D-23-02267_reviewer_coments.pdf [file pone.0284388.s007.pdf]

PLOS ONE

Inferring the age and environmental characteristics of fossil sites using citizen science

--Manuscript Draft--

**Manuscript Number:**	PONE-D-23-02267
**Article Type:**	Research Article
**Full Title:**	Inferring the age and environmental characteristics of fossil sites using citizen science
**Short Title:**	Inferring the age and environmental characteristics of fossil sites using citizen science
**Corresponding Author:**	Tara Djokic, PhD<br>Australian Museum Research Institute<br>Sydney, New South Wales AUSTRALIA
**Keywords:**	biostratigraphy; palynofossils; scanning electron microscopy; Miocene; Australia; citizen science
**Abstract:**	Not all fossil sites preserve microfossils that can be extracted using acid digestion, which may leave knowledge gaps regarding a sites age or environmental characteristics. Here we report on a citizen science approach that was developed to identify microfossils in situ on the surface of sedimentary rocks. Samples were collected from McGraths Flat, a recently discovered Miocene rainforest lake deposit located in central New South Wales, Australia. Composed entirely of iron-oxyhydroxide, McGraths Flat rocks cannot be processed using acid digestion. Scanning electron microscopy (SEM) was used to automatically acquire 25,200 high-resolution images from the surface of three McGraths Flat samples, covering a total area of 1.85 cm2. The images were published on the citizen science portal DigiVol, through which 271 citizen scientists helped to identify 300 pollen and spores. The microfossil information gained in this study is biostratigraphically relevant and can be used to constrain the environmental characteristics of McGraths Flat. Our findings suggest that automated image acquisition coupled with an evaluation by citizen scientists is an effective method of determining the age and environmental characteristics of fossiliferous rocks that cannot be investigated using acid digestion.
**Order of Authors:**	Tara Djokic, PhD
	Michael M Frese, PhD
	Adam Woods
	Mary Dettmann, PhD
	Paul Flemons
	Frank Brink, PhD
	Matthew R McCurry, PhD
**Additional Information:**	
**Question**	**Response**
**Financial Disclosure**<br><br>Enter a financial disclosure statement that describes the sources of funding for the work included in this submission. Review the submission guidelines for detailed requirements. View published research articles from *PLOS ONE* for specific examples.<br><br>This statement is required for submission and **will appear in the published article** if	M.R.M. and M.F. received funding from:<br>1. Australian Museum Foundation (no grant number issued)<br>https://australian.museum/get-involved/join/foundation/<br><br>2. The Etheridge Family Descendants (no grant number issued) - a philanthropic contribution<br>No URL available<br><br>3. An Australian Research Council (ARC) Linkage Grant (LP210301049)<br>https://www.arc.gov.au/<br><br>The funders had no role in study design, data collection and analysis, decision to

Powered by Editorial Manager® and ProduXion Manager® from Aries Systems Corporation

the submission is accepted. Please make sure it is accurate.<br><br>**Unfunded studies**<br>Enter: *The author(s) received no specific funding for this work.*<br><br>**Funded studies**<br>Enter a statement with the following details:<br>• Initials of the authors who received each award<br>• Grant numbers awarded to each author<br>• The full name of each funder<br>• URL of each funder website<br>• Did the sponsors or funders play any role in the study design, data collection and analysis, decision to publish, or preparation of the manuscript?<br>• **NO** - Include this sentence at the end of your statement: *The funders had no role in study design, data collection and analysis, decision to publish, or preparation of the manuscript.*<br>• **YES** - Specify the role(s) played.<br><br>* typeset	publish, or preparation of the manuscript.
**Competing Interests**<br><br>Use the instructions below to enter a competing interest statement for this submission. On behalf of all authors, disclose any competing interests that could be perceived to bias this work—acknowledging all financial support and any other relevant financial or non-financial competing interests.<br><br>This statement is required for submission and **will appear in the published article** if the submission is accepted. Please make sure it is accurate and that any funding sources listed in your Funding Information later in the submission form are also declared in your Financial Disclosure statement.<br><br>View published research articles from *PLOS ONE* for specific examples.	The authors have declared that no competing interests exist.

**NO authors have competing interests**<br><br>Enter: *The authors have declared that no competing interests exist.*<br><br>**Authors with competing interests**<br><br>Enter competing interest details beginning with this statement:<br><br>*I have read the journal's policy and the authors of this manuscript have the following competing interests: [insert competing interests here]*<br><br>* typeset	
**Ethics Statement**<br><br>Enter an ethics statement for this submission. This statement is required if the study involved:<br><br>• Human participants<br>• Human specimens or tissue<br>• Vertebrate animals or cephalopods<br>• Vertebrate embryos or tissues<br>• Field research<br><br>Write "N/A" if the submission does not require an ethics statement.<br><br>General guidance is provided below. Consult the submission guidelines for detailed instructions. **Make sure that all information entered here is included in the Methods section of the manuscript.**	The Human Research Ethics Committee of the University of Canberra has decided that project “11906—inferring the age and environmental characteristics of fossil sites using citizen science" is exempt from review.

Powered by Editorial Manager® and ProduXion Manager® from Aries Systems Corporation

**Format for specific study types**<br><br>**Human Subject Research (involving human participants and/or tissue)**<br>• Give the name of the institutional review board or ethics committee that approved the study<br>• Include the approval number and/or a statement indicating approval of this research<br>• Indicate the form of consent obtained (written/oral) or the reason that consent was not obtained (e.g. the data were analyzed anonymously)<br><br>**Animal Research (involving vertebrate animals, embryos or tissues)**<br>• Provide the name of the Institutional Animal Care and Use Committee (IACUC) or other relevant ethics board that reviewed the study protocol, and indicate whether they approved this research or granted a formal waiver of ethical approval<br>• Include an approval number if one was obtained<br>• If the study involved *non-human primates*, add *additional details* about animal welfare and steps taken to ameliorate suffering<br>• If anesthesia, euthanasia, or any kind of animal sacrifice is part of the study, include briefly which substances and/or methods were applied<br><br>**Field Research**<br><br>Include the following details if this study involves the collection of plant, animal, or other materials from a natural setting:<br>• Field permit number<br>• Name of the institution or relevant body that granted permission	
**Data Availability**<br><br>Authors are required to make all data underlying the findings described fully available, without restriction, and from the time of publication. PLOS allows rare exceptions to address legal and ethical concerns. See the PLOS Data Policy and FAQ for detailed information.	Yes - all data are fully available without restriction

Powered by Editorial Manager® and ProduXion Manager® from Aries Systems Corporation

A Data Availability Statement describing where the data can be found is required at submission. Your answers to this question constitute the Data Availability Statement and **will be published in the article**, if accepted.<br><br>**Important:** Stating 'data available on request from the author' is not sufficient. If your data are only available upon request, select 'No' for the first question and explain your exceptional situation in the text box.<br><br>Do the authors confirm that all data underlying the findings described in their manuscript are fully available without restriction?	
**Describe where the data may be found in full sentences. If you are copying our sample text, replace any instances of XXX with the appropriate details.**<br><br>• If the data are **held or will be held in a public repository**, include URLs, accession numbers or DOIs. If this information will only be available after acceptance, indicate this by ticking the box below. For example: *All XXX files are available from the XXX database (accession number(s) XXX, XXX.)*.<br>• If the data are all contained **within the manuscript and/or Supporting Information files**, enter the following: *All relevant data are within the manuscript and its Supporting Information files.*<br>• If neither of these applies but you are able to provide **details of access elsewhere**, with or without limitations, please do so. For example:<br><br>*Data cannot be shared publicly because of [XXX]. Data are available from the XXX Institutional Data Access / Ethics Committee (contact via XXX) for researchers who meet the criteria for access to confidential data.*<br><br>*The data underlying the results presented in the study are available from (include the name of the third party*	All raw expedition data (CSV files) and collated data can be found at:<br>https://www.dropbox.com/sh/6k6r0q19bveyaos/AACbp_8WjPuFzoi_hk3T7YC_a?dl=0 (PLEASE NOTE TO THE EDITOR THIS CAN BE CHANGED TO A MORE STABLE LOCATION)<br><br>Australian Museum 'Date a Fossil' website<br>https://australian.museum/get-involved/citizen-science/date-a-fossil/?gclid=CjwKCAiAv9ucBhBXEiwA6N8nYGOrtWeP5M4I3002y2gg9Nz2z-K1PM3m-IHwHAsoeXsnBZPB3gJnAxoConQQAvD_BwE<br>•Expeditions on DigiVol – now completed/inactive expeditions<br>(The above expedition data was exported from this)<br>https://volunteer.ala.org.au/institution/index/81538561#/expeditionList

Powered by Editorial Manager® and ProduXion Manager® from Aries Systems Corporation

and contact information or URL).<br>• This text is appropriate if the data are owned by a third party and authors do not have permission to share the data.<br><br>* typeset	
Additional data availability information:	Tick here if your circumstances are not covered by the questions above and you need the journal’s help to make your data available.

Powered by Editorial Manager® and ProduXion Manager® from Aries Systems Corporation

Manuscript
Click here to access/download;Manuscript;Djokic et al manuscript PLoS ONE.docx

Inferring the age and environmental characteristics of fossil sites using citizen science

Tara Djokic[1,2,3]*, Michael M. Frese [1,4,5], Adam Woods[1], Mary Dettmann [1,6], Paul Flemons[1], Frank Brink[7], Matthew R. McCurry[1,2,8]

1 Australian Museum Research Institute, 1 William Street, Sydney, New South Wales 2010, Australia, **2** ESSRC, School of Biological, Earth and Environmental Sciences (BEES), University of New South Wales, Kensington, New South Wales 2052, Australia, **3** School of Earth and Environmental Sciences, The University of Queensland, St Lucia, Queensland 4067 Australia, **4** Commonwealth Scientific and Industrial Research Organisation, Health and Biosecurity, Black Mountain, Australian Capital Territory 2601, Australia, **5** Faculty of Science and Technology, University of Canberra, Bruce, Australian Capital Territory 2601, Australia, **6** Geosciences, Queensland Museum, Queensland 4101, Australia, **7** Centre for Advanced Microscopy, Australian National University, Acton, ACT 2601, Australia, **8** Paleobiology, National Museum of Natural History, Smithsonian Institution, Washington, DC 20560, USA.

***t.djokic@unsw.edu.au**

Abstract

Not all fossil sites preserve microfossils that can be extracted using acid digestion, which may leave knowledge gaps regarding a sites age or environmental characteristics. Here we report on a citizen science approach that was developed to identify microfossils in situ on the surface of sedimentary rocks. Samples were collected from McGraths Flat, a recently discovered Miocene rainforest lake deposit located in central New South Wales, Australia. Composed entirely of iron-oxyhydroxide, McGraths Flat rocks cannot be processed using acid digestion. Scanning electron microscopy (SEM) was used to automatically acquire 25,200 high-resolution images from the surface of three McGraths Flat samples, covering a total area of 1.85 cm^2. The images were published on the citizen science portal DigiVol, through which 271 citizen scientists helped

to identify 300 pollen and spores. The microfossil information gained in this study is biostratigraphically relevant and can be used to constrain the environmental characteristics of McGraths Flat. Our findings suggest that automated image acquisition coupled with an evaluation by citizen scientists is an effective method of determining the age and environmental characteristics of fossiliferous rocks that cannot be investigated using acid digestion.

Keywords: biostratigraphy, palynofossils, scanning electron microscopy, Miocene, Australia, citizen science

Introduction

Citizen science is a proven method for processing large-scale biological datasets and conveying scientific approaches to a general audience [1-3]. Thus far, most biology-related citizen science projects have assessed extant biodiversity and assisted in conservation management [4-7]. However, citizen scientists have rarely been engaged in palaeontology research for reasons including that fossils can be fragile and difficult to access [8, 9]. At the time of writing, only one palaeontology project was identified from more than 3000 projects listed on the global citizen science hub, SciStarter (using search terms: ‘fossil’ and ‘palaeontology/paleontology’; https://scistarter.org/). For palaeontology to benefit from large-scale data processing, researchers will need to seek innovative ways of connecting with citizen science.

Here, we present a novel citizen science method for locating and identifying microfossils in situ from tens of thousands of scanning electron microscope (SEM) images from the surface of sedimentary rocks. Microfossils (e.g., pollen and spores) provide an important resource for determining the age and environmental characteristics of fossil sites. Traditionally, microfossils are extracted from sedimentary rocks using a variety of acid dissolution techniques [10, 11]. Acids are chosen based on their ability to dissolve the rock matrix (e.g., silicate, carbonate), but leave the organic (e.g., sporopollenin) or mineralised (e.g., silica, calcareous) microfossils intact [12]. This method is only effective if the fossils and surrounding rock matrix are compositionally different (heterogeneous) [12]. However, some sites preserve fossils and rock matrix that are compositionally similar (homogeneous) [13]. Therefore, in situ observation, rather than acid extraction, is useful to identify microfossils in chemically homogeneous sedimentary rocks.

For this study, we used rocks collected from McGraths Flat located in central New South Wales, Australia [13]. Interpreted as a Miocene age (11–20 million-years-old) rainforest lake deposit, McGraths Flat contains exceptionally well-preserved compressions/impressions of leaves, flowers and fruiting bodies, aquatic and terrestrial insects, spiders, fishes and other vertebrates (Fig. 1) [13, 14]. In addition to these macrofossils, a diverse suite of microfossils, including pollen, spores, yeast cells, nematodes, and insect wing scales are also very well-preserved [13]. All fossils and the surrounding sedimentary matrix in McGraths Flat rocks are almost entirely composed of iron-oxyhydroxide (goethite). Therefore, acid extraction methods are not possible. However, in situ microfossil observation is possible on the surface of McGraths Flat samples. Using a hammer and chisel, the samples can be split easily along relatively flat bedding planes.

Furthermore, the high conductivity of iron-rich sedimentary rocks enables high-resolution (2 nm) SEM imaging of microfossils without having to apply a conductive coating [13]. These discoveries prompted us to develop a new observational method for collecting and processing large in situ microfossil image data sets with the help of citizen scientists.

Fig. 1. Location and fossils of McGraths Flat.

(*a*) Map of Australia depicting the location of McGraths Flat (red star) near the town of Gulgong, Central Tablelands, New South Wales, Australia. (*b*) Field site. (*c*) Cross-section of the finely-bedded goethite-rich rock from McGraths Flat. (*d*) Myrtaceous (eucalypt) leaf (AM F.146592). (*e*) Budding Malvales~~(?)~~ flowers (AM F.146589). (*f*) Sawfly (Tenthredinoidea: Symphyta) (AM F.145093). Note the variation in colour (yellow-red-brown) is dominantly due to grain size, the overall composition is homogeneous. Scale bars, *c*—1 cm, *d*—1 cm, *e*—1 mm, *f*—2 mm.

There are, however, some disadvantages for in situ microfossil observation compared to acid extraction methods [11]: (*i*) Operating an SEM is time-consuming, which limits the number of microfossils that can be identified in a reasonable time frame. In 6 hrs of instrument time, a professional scientist can locate and image approximately 50 pollen and spores, which is a relatively small number compared to the hundreds to tens of thousands of specimens that can be acid extracted from as little as five grams of fine-grained clays or 50 grams of sandstone [10]; (*ii*) Rare and relatively featureless types of microfossils may be overlooked due to observation bias (e.g., large pollen with striking features are detected more readily than smaller less distinguishable specimens); and, (*iii*) Operating an SEM is relatively expensive. Besides the costs

associated with the acquisition and maintenance of the microscope, costs include the salary of a qualified specialist, usually a palaeontologist.

To address these disadvantages, we engaged citizen scientists to view, locate and identify microfossils from tens of thousands of SEM images collected using a novel automated image acquisition technique. This approach helped to process a large image dataset that can be used to determine the age and environmental characteristics of fossil deposits like McGraths Flat. We report on our findings as a case study conducted through the Australian Museum citizen science project 'Date a Fossil'.

Material and methods

(a) Microfossil image acquisition

Three relatively flat, unpolished and uncoated rock samples (AM F.146572; AM F.152543; AM F.152544) from McGraths Flat were imaged at the Centre for Advanced Microscopy located at the Australian National University, Canberra. Imaging was conducted using an FEI Quanta 650F field emission scanning electron microscope (FE-SEM) that was operated at 15 kV and with 1 nA of probe current (Fig. 2*a,b*). Samples were desiccated for 24 hrs prior to automated image acquisition to shorten the times taken to reach the minimum 3×10^{-3} Pa Torr required for high vacuum examinations. To enhance the depth of field and account for any surface irregularities in the sample, a relatively small 30 µm objective aperture was used in combination with a relatively long working distance of 15 mm. An automated image acquisition algorithm (FEI

MAPS 3.0) was used to produce image montages composed of 60 × 60 (3600) SEM images (Fig. 2*c,d*). A total of seven montages yielded an overall dataset of 25,200 SEM images. The acquisition time for each image was set to 8 seconds, resulting in a total time of approximately 8 hours to acquire a 3600-image montage. Each individual image consists of 6144 × 4090 pixels, for a 120 µm field of view, limiting the resolution to around 20 nm per pixel. An image overlap of 12.5% was used to facilitate accurate registration of each image within the montage (Fig. 2*e*). This resulted in a total imaged area of 6.3 × 4.2 mm (0.265 cm^2) for each montage. The total area imaged for all seven 60 × 60 montages (accounting for overlap) was 1.85 cm^2. Each montage was acquired from a different area across the three samples. Collecting images from different areas and samples allowed for a better representation of the microfossil density and diversity, both spatially and temporally.

Fig. 2. Automated image acquisition.

(*a*) FEI QEM SCAN 650F scanning electron microscope (SEM). (*b*) A representative rock sample (AM F.147103) from McGraths Flat (approx. 50 × 60 mm) used for automated image acquisition; the photograph was taken using the microscope chamber's digital camera. The flat surface of the rocks and their iron-rich composition makes it simple to maintain focus, as well as provide the conductivity needed for SEM operation without applying a conductive coating to the sample. (*c*) Schematic depiction of a 60 × 60 (3600) image montage; the area covered by 3600 images is 0.265 cm^2 (note that neighbouring images have a 12.5% overlap). (*d,e*) SEM images that contain microfossils: *d*—image showing a single pollen (*Nothofagidites emarcidus/heterus)*; and, *e*—two neighbouring images (dashed lines) showing an unknown spore; the images were electronically stitched together using Kolor Autopano Pro 4. The images have not been adjusted for contrast and appear in the same format as viewed by volunteers.

(b) DigiVol workflow

DigiVol is an online citizen science platform that was jointly developed by the Australian Museum, the Atlas of Living Australia and CSIRO (https://volunteer.ala.org.au/). Digivol is used to engage citizen scientists in the digitisation of natural history collections [15]. In this study, the DigiVol platform was used to find and identify microfossils (referred to as transcribing) from high-resolution SEM images in a series of online 'virtual' expeditions. SEM images were uploaded into 28 virtual expeditions on DigiVol under the project title 'Date a Fossil'. Each SEM image montage (3600 images) was divided into four expeditions (three expeditions with 1000 images each and one expedition with 600 images). This was repeated for each of the seven montages. All images in the expeditions were accompanied by a questionnaire that included illustrations (Supplementary Fig. 1). For each image, citizen scientists were asked to respond to several questions (they also had the option to skip an image). The questionnaire was designed to guide citizen scientists through a 5-step identification process (Supplementary Fig. 1):

1. Is the image out of focus? (citizen scientists answer by clicking on an image that is either *in focus* or *out of focus*).
2. Are there any microfossils in this image? (citizen scientists answer by clicking on an image *with* or *without a microfossil*).
3. How many microfossils are present? (citizen scientists answer by selecting from a drop-down list).
4. Where is the microfossil? (citizen scientists answer by clicking on an image with a microfossil or microfossils positioned in the *middle*, *edge* or both *middle and edge*).

5. What type of microfossil is present? (citizen scientists answer by clicking on one image from a selection of pollen, spores and other microfossils).

Anyone could register and participate in ‘Date a Fossil’ expeditions. The DigiVol platform is accessible via the internet and free to use (http://digivol.ala.org.au). Only one expedition was available to citizen scientists at any one time; participation in the next expedition required completion of the previous expedition.

DigiVol had two mechanisms that maintain quality assurance of captured data. The consensus approach involves an automated comparison of multiple citizen scientist inputs using a decision tree. This method is most commonly used for validating identifications of animals from camera trap images [16, 17]. Expert verification involved the manual review of validated images by an experienced scientist (i.e., project officer or palynologist).

We combined consensus and expert verification because of: (*i*) time constraints; (*ii*) pilot checking effectiveness; and, (*iii*) the intention to review data for use in further research/publication. When three citizen scientists had the same responses to the questions set out in the template, images were marked as ‘valid’. If three out of four citizen scientists did not have the same response for an image, it was listed as an ‘invalid’ image and flagged for expert review/verification. The following details the validation and verification process (Fig. 3):

- If ‘agreement’ was reached by a minimum of three citizen scientists on questions set out

in the template, the system would automatically validate the image. The two main identification categories include:

a. Agreement, fossil. In this category, all three citizen scientists agreed that at least one microfossil was present in the SEM image.
b. Agreement, no fossil. In this category, all three citizen scientists agreed that no microfossils were present in the SEM image.

- Disputed images. If a fourth citizen scientist identified a microfossil in an image *before* three others had reached 'agreement, no fossil', the disputed image would be flagged in the system. The system would still label the image with 'agreement, no fossil', but expert reviewers (project officers) would be alerted to double-check the image for microfossils.

- If 'no agreement' was reached by a minimum of three citizen scientists on questions set out in the template, the system would label the image for expert review. Microfossils that could not be easily identified by project officers were sent to a palynologist.

Similar approaches to validation (using both volunteer and expert steps) have previously been applied in camera trap studies, which effectively used two layers of quality control for data analysis [16, 17].

Fig. 3. Flow chart outlining the identification and verification steps involved in the analysis of SEM images.

Citizen scientist (volunteers) microfossil identification reduced the SEM image dataset from 25,200 to 4192. Experts reviewed the 4192 images and verified microfossils in 448 images. Accounting for image overlap, the final pollen and spore count in these verified images was 383, of which 300 specimens were identifiable. Key for superscript lettering: *a*—An 'agreement' was reached by a minimum of three volunteers on questions set out in the questionnaire template; this category was then subdivided into images indicated as having 'no microfossil/s' and images indicated as having 'microfossil/s'. *b*—These images were undisputed as having no microfossil (i.e., by an additional 4th volunteer). *c*—These images were disputed by an additional volunteer and had to be reviewed by an expert. *d*—These images were identified by three volunteers as containing other microfossils, that are not pollen or spores. *e*—If 'no agreement' was reached by a minimum of three volunteers on questions set out in the questionnaire template, these images were automatically marked by the system to be reviewed.

(c) Citizen scientist training and support

Citizen scientists were initially encouraged to read an 'identification guide' (Supplementary Fig. 2) – an instruction manual – detailing the questionnaire protocol and how to identify pollen, spores and other microfossils. The guide includes schematic representations (illustrations and SEM images) of several microfossil categories along with scientific names and size ranges. Although this guide was recommended reading, it was not mandatory for citizen scientist participation. Six weeks after 'Date a Fossil' was launched on DigiVol, an information webinar

was offered to citizen scientists. The webinar provided additional information on the project's scientific background and purpose, as well as a step-by-step guide through the identification process. Examples of microfossils and artefacts that had been recently observed by citizen scientists were displayed. Throughout the webinar, citizen scientists had the opportunity to ask questions and provide feedback. During the entire life of the project, citizen scientists were also supported on an ad hoc basis through the DigiVol online discussion forum, where they could post questions and seek technical help.

Results

The 'Date a Fossil' project was online for 3.5 months (March 15 – July 1, 2021). In that time, 271 citizen scientists participated in 28 online expeditions, analysing 25,200 SEM images. Each expedition, consisting of either 600 or 1000 images, was completed by an average of 28 citizen scientists in approximately 3 – 4 days. Each image was viewed up to four times resulting in a total of 81,939 interactions. The average time citizen scientists spent viewing and analysing an individual image was 24.9 seconds, and a total of 566 citizen scientist engagement hours were recorded. Engagement varied widely; contributions ranged from citizen scientists analysing a single image to one individual transcribing 14,509 images. Notably, 85% of the data was completed by only 10% of the transcribers. This top 10% of transcribers completed a mean number of 2565 transcriptions each. The other 90% of transcribers completed a mean number of 52 transcriptions each.

To establish the total pollen and spore count, several steps were taken throughout image processing, from volunteer validation to expert review and verification (Fig. 3; Supplementary Table 1). First, citizen scientists viewed and categorised all 25,200 images, which significantly reduced the dataset to 4192 images (Fig. 3). Project officers then reviewed the 4192 images over a total of 9 hours. Finally, any images of pollen or spores that could not be classified by project officers were sent to a palynologist for identification. This process produced the following results.

An agreement was reached on 23,612 images by three citizen scientists. These images were identified to contain either no microfossil/s (n = 23,481) or microfossil/s (n = 131). From the no microfossil group, 2473 images were disputed by a fourth citizen scientist who indicated they had observed a microfossil. A project officer reviewed the disputed images and found that only 62 images contain pollen or spores (with a total specimen count = 49). From the 131 images identified to contain microfossil/s by three citizen scientists, 98 were labelled with 'pollen or spores', and 33 were labelled 'unknown'. A review of these images by a project officer found that only 95 images contain pollen or spores (with a total specimen count = 91) and only 16 images contain unknown microfossils (Fig. 3).

No agreement was reached by three citizen scientists on 1588 images. A review by a project officer revealed pollen or spores in 275 of the 'no agreement' images (with a total specimen count = 243). Citizen scientists did not reach a consensus on the 1588 images for the following reasons: disagreement on the number of microfossils (count); whether microfossils were

present (occurrence); the identity (name) of the microfossil; and, the position of the microfossil within an image (Fig. 4). A total of 432 images contain pollen or spores. However, as a result of image overlap, only 383 specimens ~~are~~ counted. The total pollen and spore count from the entire area analysed (1.85 cm^2) includes 300 identifiable specimens and an additional 83 indeterminable specimens (Fig.s 5,6; Supplementary Table 2). The top three most abundant palynomorphs among those identified include *Nothofagidites* ssp. (44%; 72.4 specimens/cm^2), fungal spores (20%; 32.4 specimens/cm^2) and spores of ferns and mosses (9%; 15.7 specimens/cm^2). The remaining palynomorphs (27%) contribute significantly fewer population counts i.e., avg. 5.9 specimens per palynomorph/cm^2 (Fig. 5*b*; Supplementary Table 2).

To assess whether or not citizen scientists had captured/missed any significant microfossils in the dataset, a project officer manually viewed all 1000 images in Expedition No.1. It was found that seven possible pollen and spores had not been detected by citizen scientists. From this, we estimate that about 200 pollen and spores could have been overlooked across the entire dataset. Though, these specimens have indeterminable features and are therefore unidentifiable.

Fig. 4. Reasons for no agreement on images.

Venn diagram illustrating the number of times that questions related to specimen count, occurrence, position, name and image focus on the questionnaire template led to 'no agreement' among volunteer citizen scientists. The data is from 275 images that were expert verified to contain pollen or spores (see Fig. 3 for details). The top reasons that resulted in 'no agreement' included a combination of questions (count, occurrence, position and name = 78 images), followed by identification of the specimen (75

images). Occurrence—whether or not a microfossil is present in an image; count—number of pollen or spores in an image; position—placement of microfossil on the edge or middle of the image; name—selected from a range of listed specimens; and, focus—image is in focus or out of focus.

Fig. 5. Microfossil identification, counts and abundance.

(*a*) The total number of palynomorphs identified across $1.85 cm^2$ of rock surface analysed. Note 'Spores' indicate that from ferns and mosses. (*b*) Pie chart showing the relative abundance (%) of palynomorphs, and in brackets, palynomorph density (number of specimens/cm^2).

Fig. 6. Pollen and spore types identified.

(*a*) Fungal spore. (*b*) Fern spore. (*c*) *Nothofagidites*. (*d*) Gymnospermous saccate pollen. (*e*) Myrtaceae/Cupanieae pollen. (*f*) Triporate pollen. (*g*) Porate angiosperm pollen. (*h*) Araucariaceae (gymnosperm) pollen. (*i*) Angiosperm; col(por)ate pollen (*Quintinia*). (*j*) Angiosperm colporate pollen; *Margocolporites vanwijhei.* (*k*) Angiosperm colpate pollen. Dashed lines indicate the overlap/stitching between neighbouring images. Scale bars, 15 µm. Arrows indicate the position of microfossils. Images have be adjusted slightly to improve contrast. Images appearing as they were viewed by volunteers can be found in Supplementary Fig. 3.

Discussion

Time, cost and research efficiency

Citizen scientists analysed a 25,200-image dataset and identified microfossils or microfossil-like objects in 4192 images. By doing so, citizen scientists significantly reduced the initial image dataset and conducted a preliminary identification. We estimate that a trained project officer could complete an initial viewing of 25,200 images in approximately 35 hours (one working week), based on a browsing time of 5 seconds per image. In contrast, the time taken for a project officer to review a reduced dataset of 4192 images is approximately 6 hours. However, these calculations do not consider the time involved in data organisation and specific identifications (in contrast to recognising a general microfossil occurrence) or accuracy lost to observer fatigue. Taken together, it is evident that the contribution of citizen scientists can substantially reduce the time required to identify where microfossils exist within large image datasets.

Automated image acquisition and citizen scientists also reduce SEM costs and enable additional quantitative analyses. Earlier work on microfossils from McGraths Flat provided some reference points[13]. An experienced professional scientist requires a full working day (about 6 hours of instrument time) to discover and image approximately 40–50 pollen and spores. However, the scientist used a less structured approach that involved scanning the rock surface at different and constantly changing magnifications to cover as much of the rock surface as possible. Furthermore, the scientist often ignored specimens that are only partly preserved, not well

presented or belong to species that have already been imaged. Thus, although professional scientists may be better at collecting high-quality images, the citizen science approach generates much more quantitative data (Fig. 5). Furthermore, citizen scientists reduce bias through a more structured, systematic image analysis involving multiple individuals; it may also lead to the detection of smaller, less obvious or less abundant microfossils (e.g., Fig. 6*h,i*).

Accuracy and improvements in citizen scientist microfossil identification

Our approach of using a multi-staged identification system (citizen science identification and expert review) was successful in accurately capturing most, if not all, pollen and spores in a big image dataset. The expert review of all 1000 images in Expedition No. 1, revealed that only a few (i.e., seven) indeterminable 'possible' pollen and spores were undetected by citizen scientists. However, the main aims of this study are unaffected because these undetected specimens have indeterminable characteristics.

A frequent lack of agreement among citizen scientists concerning whether or not an image exhibited pollen or spore/s suggests that there is room for improvement in our approach. Approximately 70–75% of pollen and spores were only identified through the second tier of citizen scientist assessment (i.e., by evaluating 'disputed images') or by expert review of images that had been labelled with 'no agreement' (Fig. 3). Upon expert review, however, only a small proportion of images (18% or 289 out of 1588 images) for which 'no agreement', among three citizen scientists, was reached were found to contain pollen or spores. Likewise, upon expert review of 'disputed' images, in which a fourth citizen scientist disputed the 'agreement' of

three citizen scientists, only 2% (or 62 out of 2473 images) were found to contain pollen or spores. These shortcomings can be addressed easily by changing the questionnaire. Removal of unnecessary questions (e.g., the fossil position or the number of fossils; Fig. 4) is likely to increase the level of agreement among citizen scientists. Providing better citizen scientist training (e.g., through online workshops or more comprehensive teaching material) may further improve accuracy in name identification. These findings are in line with other citizen science projects that analysed a large number of SEM images [8].

Implications for palaeontology research

Microfossils provide valuable information about the age, fossilisation processes and palaeoenvironmental characteristics of fossil sites [13, 18-20]. In this study, citizen scientists successfully helped to identify over 300 fossil pollen and spore specimens from rock surfaces. Automated image acquisition allowed us to determine the relative microfossil abundance and palynomorph density/cm^2 (Fig. 5), and promoted the discovery of significant specimens through a structured approach. The microfossils identified here include rare or difficult to identify pollen such as Araucariaceae (gymnosperm) pollen and angiosperm, col(por)ate pollen (*Quintinia*) (Fig. 6*h,i*). Furthermore, automated image acquisition allows for better use of instrument time; in our case, the SEM was operated overnight and on weekends. Professionals who employ a less structured approach are likely to overlook less obvious taxa. The comprehensive palynological dataset acquired in this study can be used to constrain the age and reconstruct the environmental characteristics of McGraths Flat [13].

These findings have implications for in situ image investigation in other fossil sites. Automatic image acquisition coupled with citizen scientists could be applied to any fossiliferous rocks that can be imaged with reasonable resolution. Silicified fossil deposits such as the Devonian Rhynie Chert of Scotland [21, 22], or the Siderian Turee Creek Group of Western Australia [23] can not yield microfossils through acid digestion but contain abundant and diverse microfossils observable in situ through SEM or optical microscopy. Petrographic image analysis of microfossils could be carried out by citizen scientists as has been done with SEM images. Likewise, processing of other imagery (e.g., aerial photographs of fossil trackways; [24]) is possible with our approach, given that citizen scientists are guided and that automated image acquisition technology is available.

It is hoped that studies such as ours may stimulate the development of more advanced microscope and imaging software technology e.g., improved auto-focusing of non-polished and thus uneven rock surfaces, image acquisition speed, image resolution and more user-friendly data managing software. Large datasets could also be processed using machine learning. Such an approach has already been implemented to locate and classify extant pollen [25] and to reconstruct palaeoclimates using stomatal density [25, 26]. A downside to a machine learning approach may be that it removes the opportunity for palaeontologists to engage the public in scientific practice. However, validated microfossil image databases are needed to train machine learning algorithms [25, 27]. Therefore, a coupled workflow involving citizen science and machine learning could benefit palaeontological research *and* recruit and train citizen scientists [28].

Conclusion

The 'Date a Fossil' project successfully engaged with citizen scientists to identify microfossils in situ on rock surfaces. Citizen scientists were exposed to highly fossiliferous rocks from a Miocene fossil site (McGraths Flat) that contained a diverse microfossil assemblage (pollen, spores, protists, bacteria and plant and animal fragments). Automated SEM image acquisition generated 25,200 images in which 300 pollen and spores were identified. Significantly, the age and environmental characteristics of McGraths Flat could be determined with the identified palynological data. We hope that our results inspire other scientists to use automated imaging to scan the surfaces of rocks that cannot be studied using acid digestion techniques. Finally, our methods could be applied to other research that seeks to process a variety of large in situ image datasets, while engaging and training the public in palaeontology.

References

1. Bonney R, Cooper CB, Dickinson J, Kelling S, Phillips T, Rosenberg KV, et al. Citizen science: a developing tool for expanding science knowledge and scientific literacy. BioScience. 2009;59(11): 977-84.
2. Miller-Rushing A, Primack R, Bonney R. The history of public participation in ecological research. Frontiers in Ecology and the Environment. 2012;10(6): 285-90.
3. Gura T. Citizen science: amateur experts. Nature. 2013;496(7444): 259-61.
4. Chandler M, See L, Copas K, Bonde AM, López BC, Danielsen F, et al. Contribution of citizen science towards international biodiversity monitoring. Biological conservation. 2017;213: 280-94.
5. Jones FM, Allen C, Arteta C, Arthur J, Black C, Emmerson LM, et al. Time-lapse imagery and volunteer classifications from the Zooniverse Penguin Watch project. Scientific data. 2018;5(1): 1-13.

6. Callaghan CT, Rowley JJ, Cornwell WK, Poore AG, Major RE. Improving big citizen science data: Moving beyond haphazard sampling. PLoS Biology. 2019;17(6): e3000357.
7. Rowley JJ, Callaghan CT, Cornwell WK. Widespread short-term persistence of frog species after the 2019–2020 bushfires in eastern Australia revealed by citizen science. Conservation Science and Practice. 2020;2(11): e287.
8. Soul LC, Barclay RS, Bolton A, Wing SL. Fossil Atmospheres: a case study of citizen science in question-driven palaeontological research. Philosophical Transactions of the Royal Society B. 2019;374(1763): 20170388.
9. Perez VJ, Leder RM, Lundgren L, Ellis S, Dunckel BA, Crippen KJ. The Belgrade PaleoBlitz: A pilot project to engage amateur paleontologists. Palaeontologica Electronica. 2020;23(1): a18.
10. Phipps D, Playford G. Laboratory techniques for extraction of palynomorphs from sediments. Papers of the Department of Geology of the University of Queensland. 1984;2(1): 1-23.
11. Traverse A. Paleopalynology. Boston: Unwim Hyman; 1988. 600pp p.
12. Wood GD, Gabriel, A.M. & Lawson, J.C. Palynological techniques - processing and microscopy. In: Jansonius JM, D.C, editor. In: Palynology: principles and applications. Salt Lake City: American Association of Stratigraphic Palynologists Foundation. Publishers Press; 1996. p. 29–50.
13. McCurry MR, Cantrill DJ, Smith PM, Beattie R, Dettmann M, Baranov V, et al. A Lagerstätte from Australia provides insight into the nature of Miocene mesic ecosystems. Science advances. 2022;8(1): e1406.
14. Moulds M, Frese M, McCurry M. New cicada fossils from Australia (Hemiptera: Cicadoidea: Cicadidae) with remarkably detailed wing surface nanostructure. Alcheringa: An Australasian Journal of Palaeontology. 2022: 1-13.
15. Flemons P, Berents P. Image based Digitisation of Entomology Collections: Leveraging volunteers to increase digitization capacity. ZooKeys. 2012(209): 203-17.
16. Swanson A, Kosmala M, Lintott C, Simpson R, Smith A, Packer C. Snapshot Serengeti, high-frequency annotated camera trap images of 40 mammalian species in an African savanna. Scientific data. 2015;2(1): 1-14.
17. Hsing PY, Bradley S, Kent VT, Hill RA, Smith GC, Whittingham MJ, et al. Economical crowdsourcing for camera trap image classification. Remote Sensing in Ecology and Conservation. 2018;4(4): 361-74.
18. Coles G, Gilbertson D, Hunt C, Jenkinson R. Taphonomy and the palynology of cave deposits. Cave Science. 1989;16(3): 83-9.
19. Dunn K, McLean R, Upchurch Jr G, Folk R. Enhancement of leaf fossilization potential by bacterial biofilms. Geology. 1997;25(12): 1119-22.
20. Macphail MK. Palynostratigraphy of the Murray Basin, inland southeastern Australia. Palynology. 1999;23(1): 197-240.
21. Strullu-Derrien C, Kenrick P, Knoll AH. The Rhynie chert. Current Biology. 2019;29(23): R1218-R23.
22. Wellman CH, Kerp H, Hass H. Spores of the Rhynie chert plant Aglaophyton (Rhynia) major (Kidston and Lang) DS Edwards, 1986. Review of Palaeobotany and Palynology. 2006;142(3-4): 229-50.

23. Barlow EV, Van Kranendonk MJ. Snapshot of an early Paleoproterozoic ecosystem: Two diverse microfossil communities from the Turee Creek Group, Western Australia. Geobiology. 2018;16(5): 449-75.
24. Bibi F, Kraatz B, J Beech M, Hill A. Fossil trackways of the Baynunah Formation. Sands of Time: Springer; 2022. p. 283-98.
25. Holt K, Allen G, Hodgson R, Marsland S, Flenley J. Progress towards an automated trainable pollen location and classifier system for use in the palynology laboratory. Review of Palaeobotany and Palynology. 2011;167(3-4): 175-83.
26. France I, Duller AWG, Duller GAT, Lamb HF. A new approach to automated pollen analysis. Quaternary Science Reviews. 2000;19(6): 537-46.
27. Fetter KC, Eberhardt S, Barclay RS, Wing S, Keller SR. StomataCounter: a neural network for automatic stomata identification and counting. New Phytologist. 2019;223(3): 1671-81.
28. Renaudie J, Lazarus DB, Diver P. NSB (Neptune Sandbox Berlin): an expanded and improved database of marine planktonic microfossil data and deep-sea stratigraphy. Palaeontol Electron. 2020;23: a11.

Data Files and other relevant material

All raw expedition data (CSV files) and collated data can be found at:
https://www.dropbox.com/sh/6k6r0q19bveyaos/AACbp_8WjPuFzoi_hk3T7YC_a?dl=0

Australian Museum 'Date a Fossil' website
https://australian.museum/get-involved/citizen-science/date-a-fossil/?gclid=CjwKCAiAv9ucBhBXEiwA6N8nYGOrtWeP5M4I3002y2gg9Nz2z-K1PM3m-IHwHAsoeXsnBZPB3gJnAxoConQQAvD_BwE

- Expeditions on DigiVol – now completed/inactive expeditions
  (The above expedition data was exported from this)
  https://volunteer.ala.org.au/institution/index/81538561#/expeditionList

Ethics approval

The Human Research Ethics Committee of the University of Canberra has decided that project "11906—inferring the age and environmental characteristics of fossil sites using citizen science" is exempt from review.

Acknowledgements

We are grateful to the Etheridge family descendants who funded this work. We thank the Australian Museum Research Institute and the Australian Museum Trust for additional funding support. We thank the Atlas of Living Australia (ALA), which receives support from the Australian Government through the National Collaborative Research Infrastructure Strategy

(NCRIS) and is hosted by the Commonwealth Scientific and Industrial Research Organisation (CSIRO). We thank Nigel McGrath for access to the fossil site. We acknowledge the traditional custodians of the land and waterways on which the McGraths Flat deposit is located, the Wiradjuri Nation People.

Funding Sources

This work was supported by grants from the Australian Museum Foundation and the Etheridge Family Descendants, as well as an ARC Linkage Grant (LP210301049).

Author contributions

The use of citizen science and automated image acquisition in this methodology was conceived by M.R.M and M.F. Automated SEM image acquisition was carried out by F.B. The DigiVol workflow was designed by M.R.M., M.F., P.F., A.W. and managed by A.W. Image data was collected and processed by T.D. Palynological expertise was provided by M.D. All authors contributed to discussion, interpretation and writing.

List of Figures

Fig. 1. Location and fossils of McGraths Flat. (*a*) Map of Australia depicting the location of McGraths Flat (red star) near the town of Gulgong, Central Tablelands, New South Wales, Australia. (*b*) Field site. (*c*) Cross-section of the finely-bedded goethite-rich rock from McGraths Flat. (*d*) Myrtaceous (eucalypt) leaf (AM F.146592). (*e*) Budding Malvales(?) flowers (AM F.146589). (*f*) Sawfly (Tenthredinoidea: Symphyta) (AM F.145093). Note the variation in colour (yellow-red-brown) is dominantly due to grain size, the overall composition is homogeneous. Scale bars, *c*—1 cm, *d*—1 cm, *e*—1 mm, *f*—2 mm.

Fig. 2. Automated image acquisition. (*a*) FEI QEM SCAN 650F scanning electron microscope (SEM). (*b*) A representative rock sample (AM F.147103) from McGraths Flat (approx. 50 × 60 mm) used for automated image acquisition; the photograph was taken using the microscope chamber's digital camera. The flat surface of the rocks and their iron-rich composition makes it simple to maintain focus, as well as provide the conductivity needed for SEM operation without applying a conductive coating to the sample. (*c*) Schematic depiction of a 60 × 60 (3600) image montage; the area covered by 3600 images is 0.265 cm^2 (note that neighbouring images have a 12.5% overlap). (*d,e*) SEM images that contain microfossils: *d*—image showing a single pollen (*Nothofagidites emarcidus/heterus)*; and, *e*—two neighbouring images (dashed lines) showing an unknown spore; the images were electronically stitched together using Kolor Autopano Pro 4. The images have not been adjusted for contrast and appear in the same format as viewed by volunteers.

Fig. 3. Flow chart outlining the identification and verification steps involved in the analysis of SEM images. Citizen scientist (volunteers) microfossil identification reduced the SEM image dataset from 25,200 to 4192. Experts reviewed the 4192 images and verified microfossils in 448 images. Accounting for image overlap, the final pollen and spore count in these verified images was 383, of which 300 specimens were identifiable. Key for superscript lettering: *a*—An 'agreement' was reached by a minimum of three volunteers on questions set out in the questionnaire template; this category was then subdivided into images indicated as having 'no microfossil/s' and images indicated as having

'microfossil/s'. *b*—These images were undisputed as having no microfossil (i.e., by an additional 4th volunteer). *c*—These images were disputed by an additional volunteer and had to be reviewed by an expert. *d*—These images were identified by three volunteers as containing other microfossils, that are not pollen or spores. *e*—If 'no agreement' was reached by a minimum of three volunteers on questions set out in the questionnaire template, these images were automatically marked by the system to be reviewed.

Fig. 4. Reasons for no agreement on images. Venn diagram illustrating the number of times that questions related to specimen count, occurrence, position, name and image focus on the questionnaire template led to 'no agreement' among volunteer citizen scientists. The data is from 275 images that were expert verified to contain pollen or spores (see Fig. 3 for details). The top reasons that resulted in 'no agreement' included a combination of questions (count, occurrence, position and name = 78 images), followed by identification of the specimen (75 images). Occurrence—whether or not a microfossil is present in an image; count—number of pollen or spores in an image; position—placement of microfossil on the edge or middle of the image; name—selected from a range of listed specimens; and, focus—image is in focus or out of focus.

Fig. 5. Microfossil identification, counts and abundance. (*a*) The total number of palynomorphs identified across 1.85cm^2 of rock surface analysed. Note 'Spores' indicate that from ferns and mosses. (*b*) Pie chart showing the relative abundance (%) of palynomorphs, and in brackets, palynomorph density (number of specimens/cm^2).

Fig. 6. Pollen and spore types identified. (*a*) Fungal spore. (*b*) Fern spore. (*c*) *Nothofagidites*. (*d*) Gymnospermous saccate pollen. (*e*) Myrtaceae/Cupanieae pollen. (*f*) Triporate pollen. (*g*) Porate angiosperm pollen. (*h*) Araucariaceae (gymnosperm) pollen. (*i*) Angiosperm; col(por)ate pollen (*Quintinia*). (*j*) Angiosperm colporate pollen; *Margocolporites vanwijhei.* (*k*) Angiosperm colpate pollen. Dashed lines indicate the overlap/stitching between neighbouring images. Scale bars, 15 µm. Arrows indicate the position of microfossils. Images have be adjusted slightly to improve contrast. Images appearing as they were viewed by volunteers can be found in Supplementary Fig. 3.

List of Supplementary Figures

Supplementary Fig. 1. Screenshots of the questionnaire template for the 'Date a Fossil Project' hosted on DigiVol.

Supplementary Fig. 2. Training manual and microfossil identification guide for citizen scientists engaging with 'Date a Fossil' on DigiVol.

Supplementarv Fig. 3. Edited (a-k) and unedited (a-k) micrographs of pollen and spore examples from McGraths Flat. At the centre is Fig. 6 from the manuscript, which is surrounded by outset images of the corresponding original unedited SEM micrographs that were viewed by citizen scientists. (a) Fungal spore. (b) Fern spore. (c) Nothofagidites. (d) Gymnospermous saccate pollen. (e) Mvrtaceae/Cupanieae pollen. (f) Triporate pollen. (g) Porate angiosperm pollen. (h) Araucariaceae (eymnosperm) pollen. (i) Angiosperm; col(por)ate pollen (Quintinia). (i) Aneiosperm colporate pollen; Margocolporites vanwihei. (k) Angiosperm colpate pollen. Dashed lines indicate the overlap/stitching between images. Arrows indicate the microfossils. Scale bars, 15 um. Field of view for all outset unedited images, 120 um.

List of Supplementary Tables

Supplementary Table 1. Breakdown of total number of images transcribed by volunteers and expert reviewed. Total number of images in which volunteers reached agreement or did not reach agreement on the questionnaire template. Total number of images that needed expert review, as well as images verified to contain pollen/spores and final pollen/spore counts.

Supplementary Table 2. Total pollen and spore counts (expert verified) from 25,200 SEM images analysed by citizen scientists and palynomorph density/cm^2.

Figure 1

Click here to access/download;Figure;Fig1.tiff

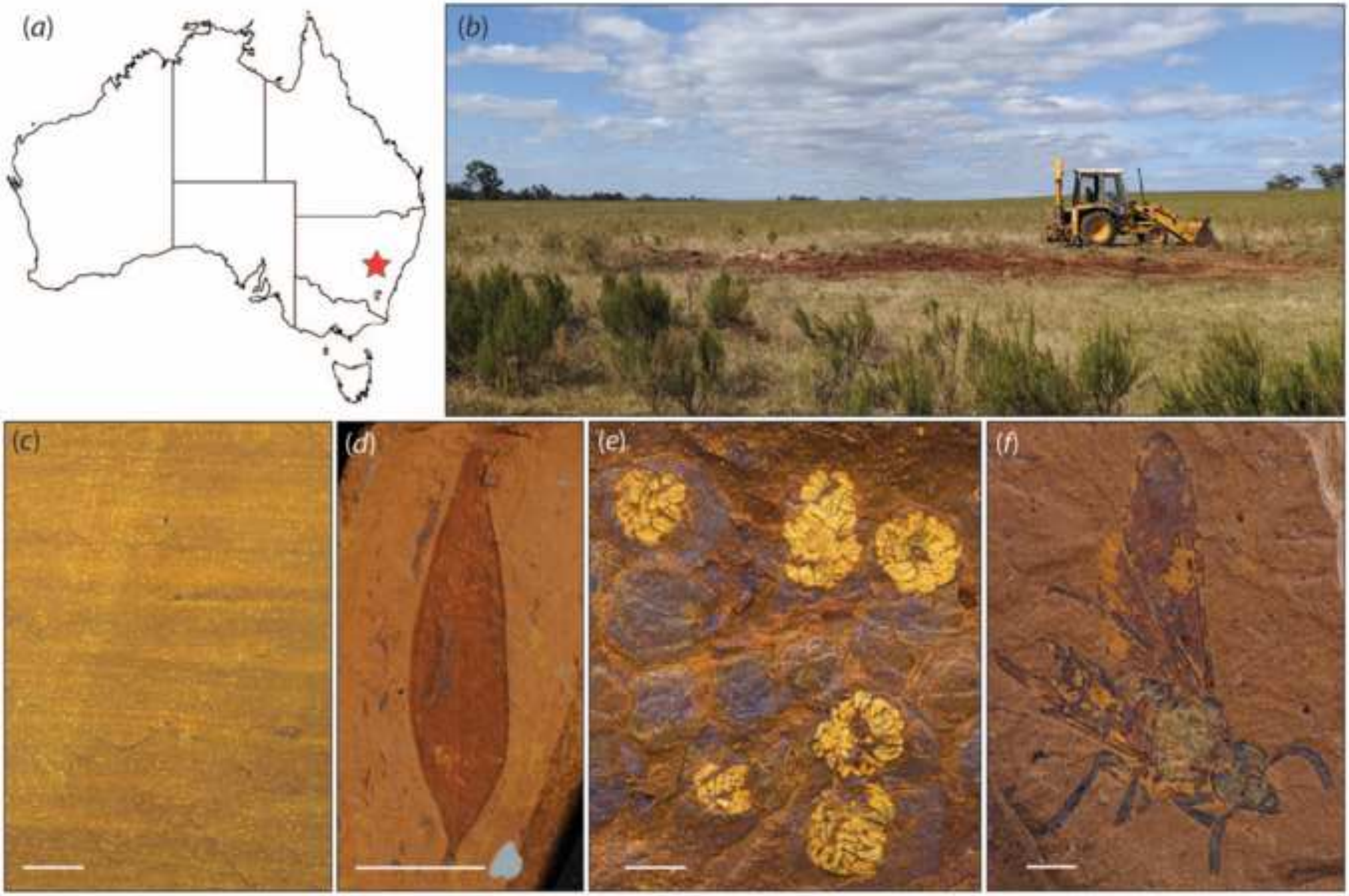

Figure 2

Click here to access/download;Figure;Fig2.tiff

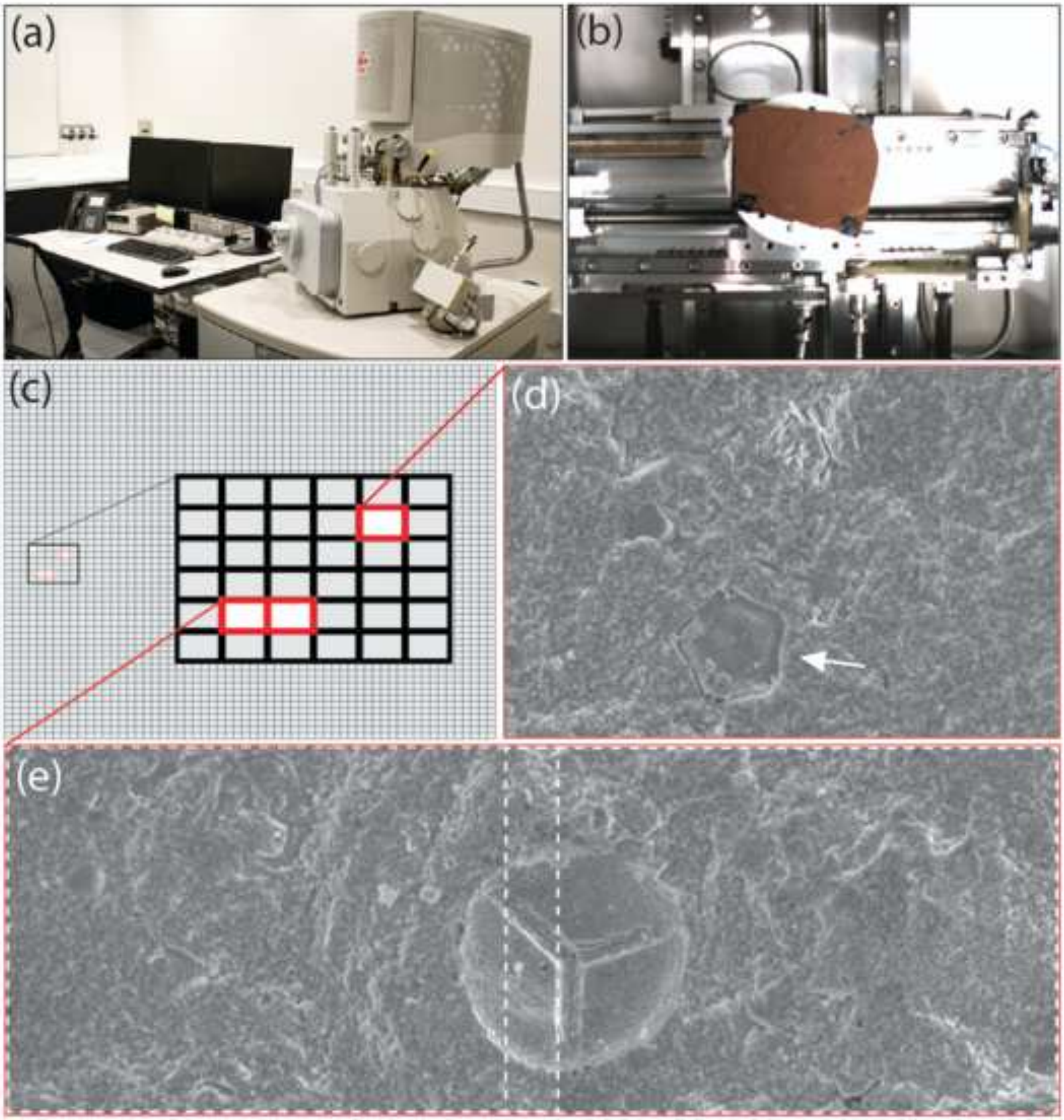

Figure 3

Click here to access/download;Figure;Fig3.tiff

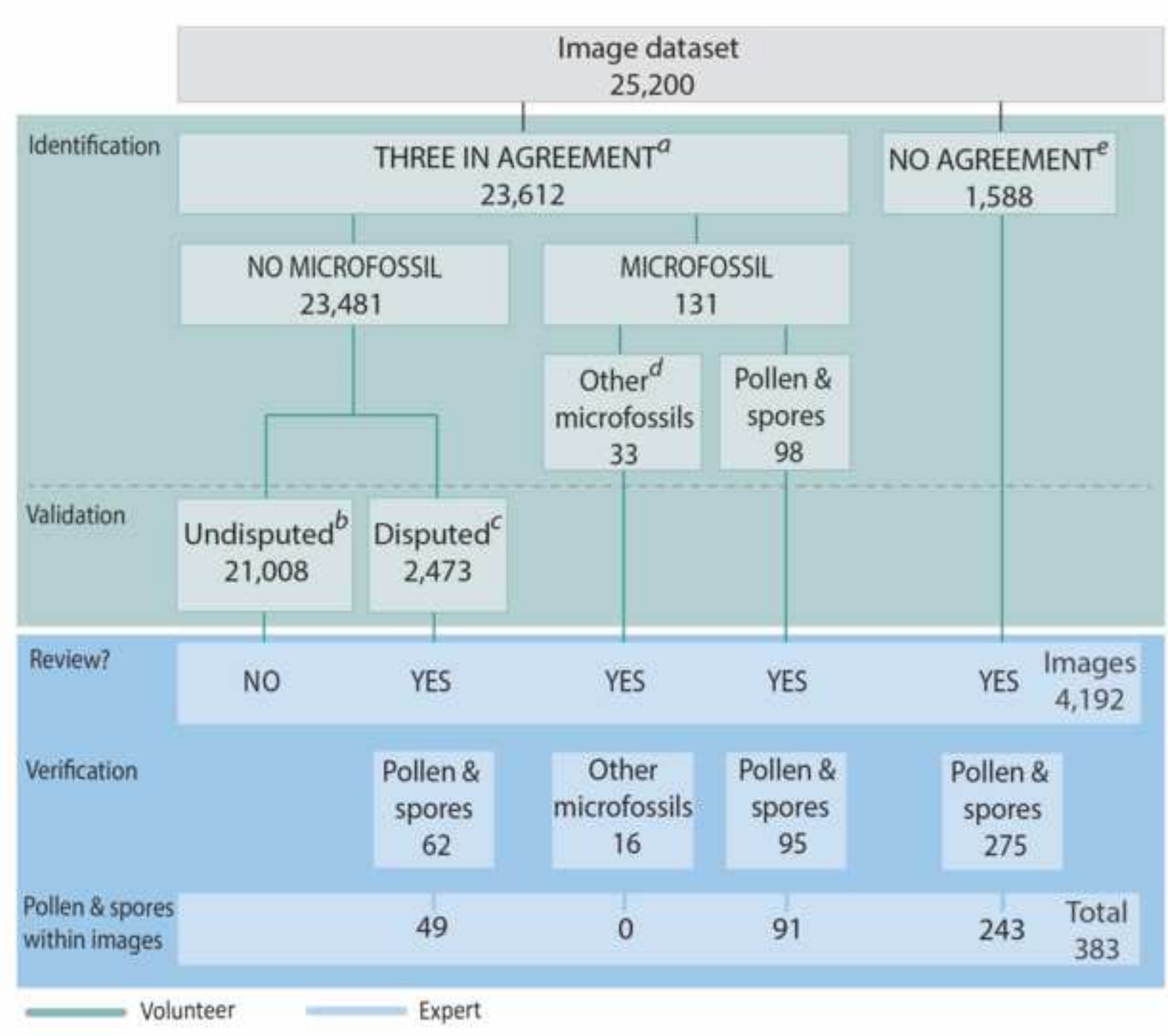

Figure 4

Click here to access/download;Figure;Fig4.tiff

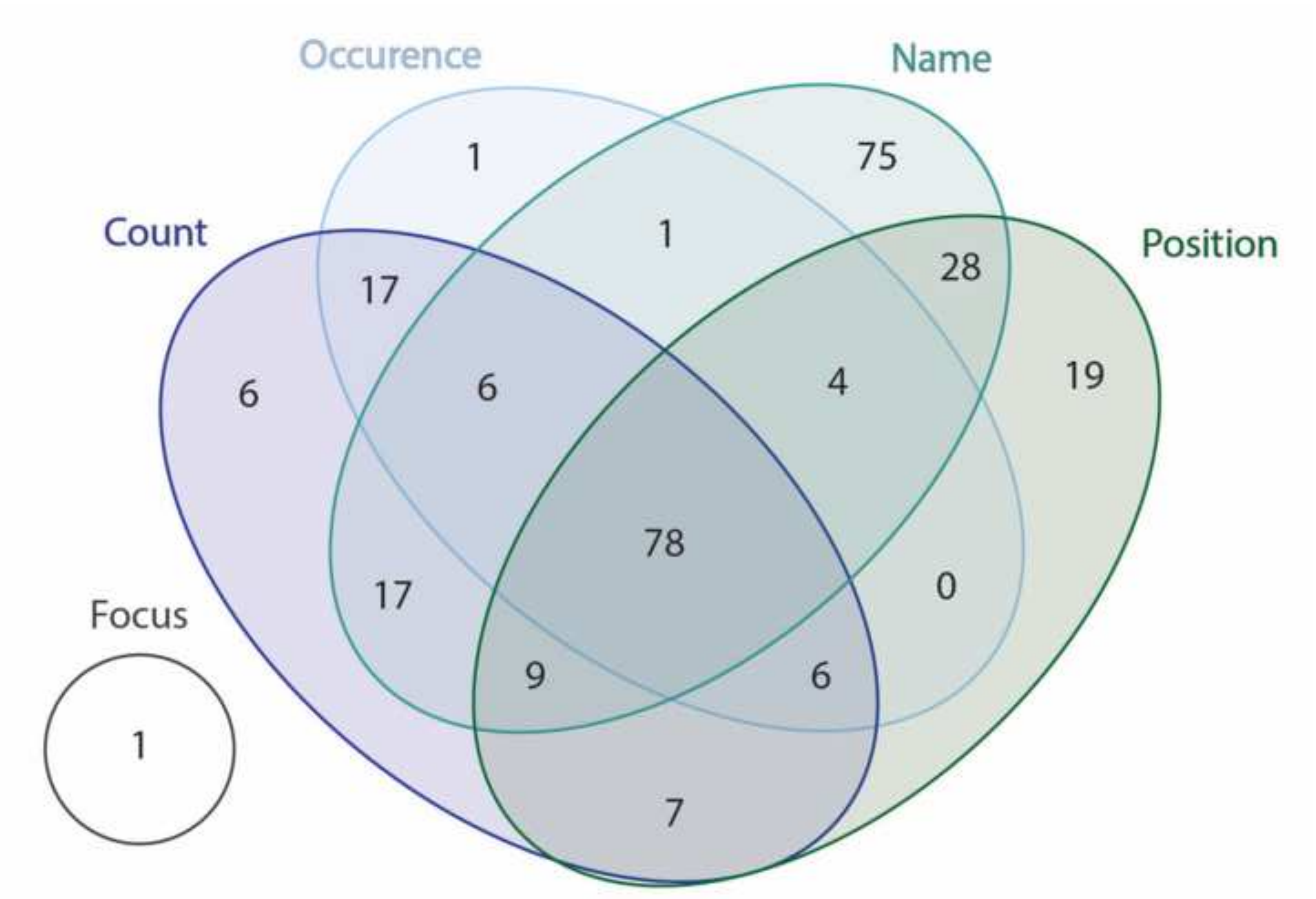

Figure 5

Click here to access/download;Figure;Fig5.tiff

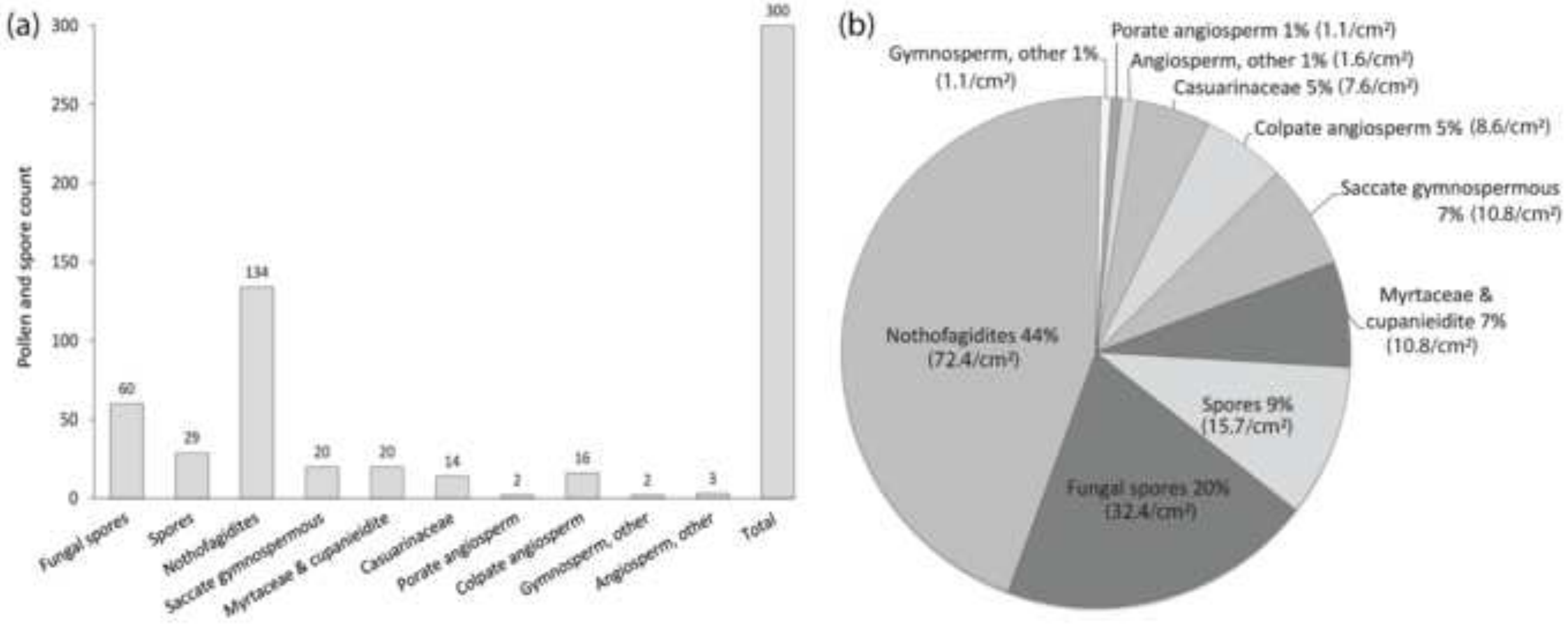

Figure 6

Click here to access/download;Figure;Fig6.tiff

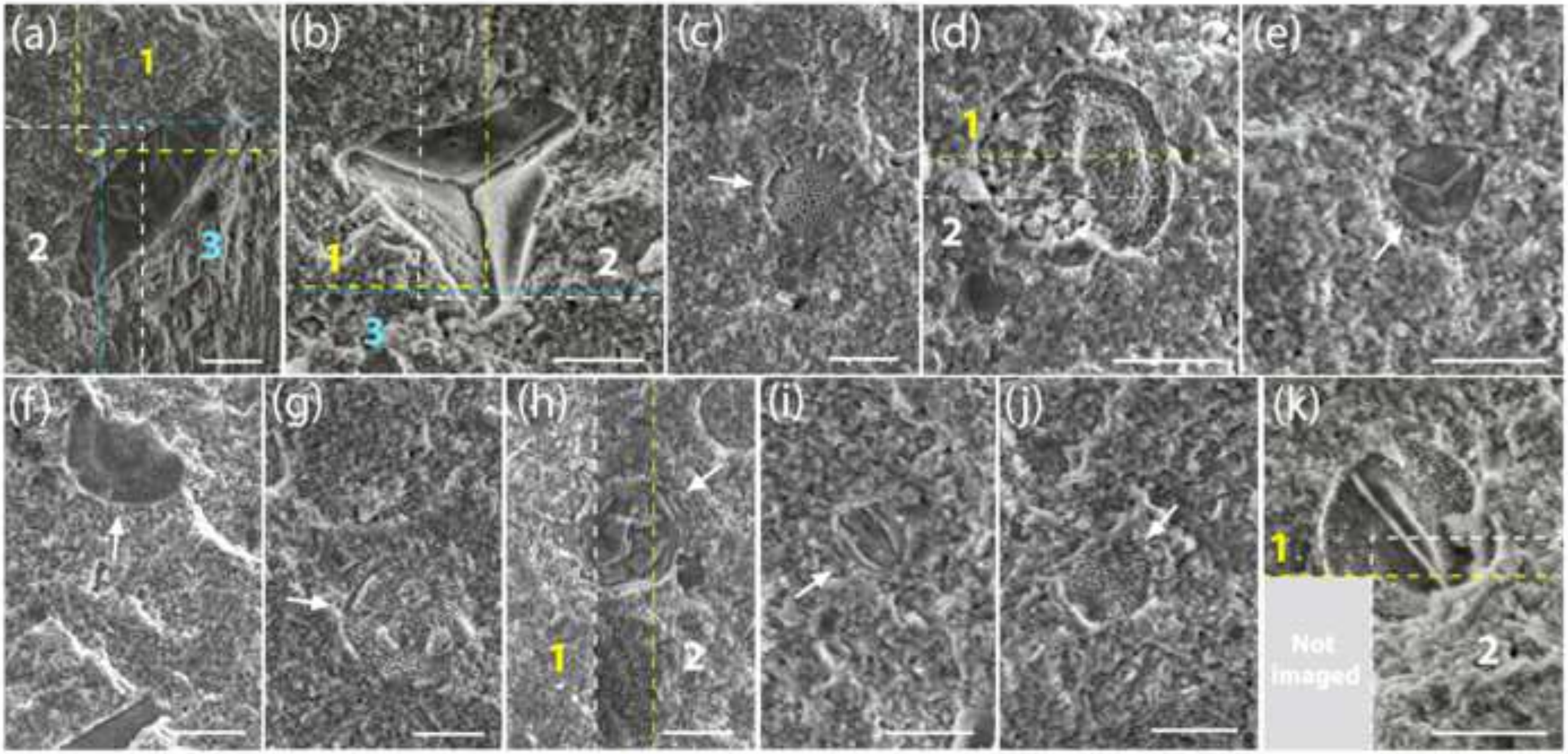

Supp. Fig. 1

Click here to access/download
Supporting Information
Supp Figure 1. questionnaire.pdf

Supp. Fig. 2

Click here to access/download
Supporting Information
Supp Figure 2. ID Manual.pdf

Supp. Fig. 3

Click here to access/download
Supporting Information
Supp Figure 3.pdf

Supp. Table 1

Click here to access/download
Supporting Information
Supp Table 1.pdf

Supp. Table 2

Click here to access/download
Supporting Information
Supp Table 2.pdf
